# Fine-tuning of mTOR signaling by the UBE4B-KLHL22 E3 ubiquitin ligase cascade in brain development

**DOI:** 10.1242/dev.201286

**Published:** 2022-12-20

**Authors:** Xiangxing Kong, Xin Shu, Jiachuan Wang, Dandan Liu, Yingchun Ni, Weiqi Zhao, Lebo Wang, Zhihua Gao, Jiadong Chen, Bing Yang, Xing Guo, Zhiping Wang

**Affiliations:** ^1^ Department of Neurobiology and Department of Neurology of Second Affiliated Hospital, NHC and CAMS Key Laboratory of Medical Neurobiology, Zhejiang University School of Medicine, Hangzhou 310058, China; ^2^ The MOE Frontier Science Center for Brain Research and Brain-Machine Integration, Zhejiang University School of Brain Science and Brain Medicine, Hangzhou 310058, China; ^3^ Zhejiang Provincial Key Laboratory for Cancer Molecular Cell Biology, Life Sciences Institute, Zhejiang University, Hangzhou, Zhejiang 310058, China; ^4^ Zhejiang University-University of Edinburgh Institute, Zhejiang University, Haining 314400, China; ^5^ Deanery of Biomedical Sciences, College of Medicine and Veterinary Medicine, University of Edinburgh, Edinburgh, EH8 9YL, UK

**Keywords:** UBE4B E3/E4 ubiquitin ligase, KLHL22, mTOR signaling, Neural precursor cells, Neurogenesis

## Abstract

Spatiotemporal regulation of the mechanistic target of rapamycin (mTOR) pathway is pivotal for establishment of brain architecture. Dysregulation of mTOR signaling is associated with a variety of neurodevelopmental disorders. Here, we demonstrate that the UBE4B-KLHL22 E3 ubiquitin ligase cascade regulates mTOR activity in neurodevelopment. In a mouse model with UBE4B conditionally deleted in the nervous system, animals display severe growth defects, spontaneous seizures and premature death. Loss of UBE4B in the brains of mutant mice results in depletion of neural precursor cells and impairment of neurogenesis. Mechanistically, UBE4B polyubiquitylates and degrades KLHL22, an E3 ligase previously shown to degrade the GATOR1 component DEPDC5. Deletion of UBE4B causes upregulation of KLHL22 and hyperactivation of mTOR, leading to defective proliferation and differentiation of neural precursor cells. Suppression of KLHL22 expression reverses the elevated activity of mTOR caused by acute local deletion of UBE4B. Prenatal treatment with the mTOR inhibitor rapamycin rescues neurogenesis defects in *Ube4b* mutant mice. Taken together, these findings demonstrate that UBE4B and KLHL22 are essential for maintenance and differentiation of the precursor pool through fine-tuning of mTOR activity.

## Teaser

**Highlighted Article:** The UBE4B-KLHL22 E3 cascade tightly controls activation of mTOR signaling and sustains perinatal neurogenesis at an appropriate level during mouse brain development.

## INTRODUCTION

In mammalian brains, sophisticated regulatory mechanisms have evolved to ensure precise establishment of the brain architecture (Paridaen and Huttner, 2014).The mechanistic target of rapamycin (mTOR) pathway orchestrates a series of events during embryonic and postnatal development, such as self-renewal and differentiation of neural stem cells (NSCs), neurite outgrowth, synaptogenesis, and synaptic remodeling (Crino, 2015; Liu and Sabatini, 2020; Parenti et al., 2020). As a highly conserved serine/threonine kinase, mTOR integrates inputs from growth factors and nutrient status through two distinct complexes (mTORC1 and mTORC2). Activation of mTORC1 initiates biosynthetic processes to support anabolism and cell growth, whereas mTORC2 governs cytoskeletal dynamics and cell survival. The activation of mTORC1 is subjected to exquisite control by a regulatory network converging on two GTPases, Rag and Rheb (Inoki et al., 2003a; Kim et al., 2008; Valvezan and Manning, 2019). The GTPase-activating protein (GAP) complex GATOR1, consisting of DEPDC5, NPRL2 and NPRL3, negatively regulates Rag (Bar-Peled et al., 2013). GTP loading of Rheb is inhibited by the tuberous sclerosis complex (TSC), which acts as a GAP and senses growth factors and environmental stress (Inoki et al., 2003b; Kim et al., 2008; Menon et al., 2014).

Given the central role of mTOR in coordinating developmental cues and growth conditions, dysregulation of mTOR and its upstream regulators can cause disastrous neurodevelopmental disorders (Crino, 2015; Liu and Sabatini, 2020; Parenti et al., 2020). Human genetic studies have identified numerous variants in genes encoding components of the mTOR pathway (Parenti et al., 2020). These mutations often lead to mTOR hyperactivation and cause a variety of symptoms, including cortical malformations, epilepsy, autism spectrum disorder and intellectual disabilities. For example, loss-of-function mutations of the mTORC1 suppressor DEPDC5 have been linked to focal cortical dysplasia-associated epilepsy (Dibbens et al., 2013; Ribierre et al., 2018). Mouse models with neuronal loss of DEPDC5 exhibit constitutive mTORC1 activation and increased phosphorylation of the downstream ribosomal protein S6 (RPS6) (Yuskaitis et al., 2018; De Fusco et al., 2020; Parenti et al., 2020). Germline or mosaic loss-of-function mutations of TSC1 or TSC2 give rise to a multisystem genetic disorder called tuberous sclerosis. Characteristic manifestations of this disorder include cortical tubers, intellectual disabilities, autism spectrum disorder and early-onset seizures (Curatolo et al., 2015). Conditional deletion of TSC1 or TSC2 in mice causes TOR hyperactivation, impaired memory, social deficits and spontaneous seizures (Ehninger et al., 2008; Crowell et al., 2015). Abnormal proliferation and differentiation of NSCs are commonly observed in these mouse models. Cultured cells often maintain high mTOR activity owing to ambient nutrients and growth factors. In contrast, cells *in vivo* tend to display lower baseline mTOR activities with sharper fluctuations to fulfill spatiotemporal requirements of organismal growth (Liu and Sabatini, 2020). Thus, it is of particular importance to investigate how mTOR is tightly regulated during brain development, an enormously complex process, at the organismal level.

In this study, we have identified the ubiquitination factor E4B (UBE4B) and KLHL22 as regulators of mTOR activity in the developing mouse brain. In cancer cells, KLHL22 degrades DEPDC5 and releases inhibition of GATOR1 on mTORC1 in response to amino acid availability (Chen et al., 2018). However, whether this regulatory mechanism applies to the nervous system and how this E3 ligase itself is regulated remain largely unknown. UBE4B is a U-box type E3/E4 ubiquitin ligase involved in multiubiquitin chain assembly (Koegl et al., 1999; Hatakeyama et al., 2001; Kaneko et al., 2003). As an E4 ligase, UBE4B binds to the ubiquitin moieties on substrates initially attached by other E3 ligases, promotes ubiquitin transfer from E2, and catalyzes elongation of polyubiquitin chains (Koegl et al., 1999; Matsumoto et al., 2004). In some cases, UBE4B can independently act as an E3 ligase and mediate polyubiquitylation by itself (Hatakeyama et al., 2001). Studies in *Caenorhabditis elegans* have demonstrated that the worm homolog UFD-2 is involved in DNA repair, stress response and proteostasis maintenance (Hoppe et al., 2004; Janiesch et al., 2007; Ackermann et al., 2016; Hellerschmied et al., 2018). In cancer studies, UBE4B promotes the degradation of the tumor suppressor p53 (TRP53), thus inhibiting cell apoptosis and promoting tumorigenesis (Wu et al., 2011; Antoniou et al., 2019). In animal models of spinocerebellar ataxia type 3 (SCA3) and Alzheimer's disease (AD), UBE4B facilitates clearance of ataxin 3 and APP, respectively (Matsumoto et al., 2004; Gireud-Goss et al., 2020). These findings have begun to unveil the important roles of UBE4B under different disease conditions. However, little is known about its physiological functions, especially during brain development. The human *UBE4B* gene is expressed in fetal and adult brains (Hosoda et al., 2005). Haploinsufficiency of human *UBE4B* has been considered as a contributing factor to the chromosomal 1p36 deletion syndrome, characterized by developmental delay, intellectual disability, seizures, cardiomyopathy, and various facial features (Jordan et al., 2015; Antoniou et al., 2019). Mouse UBE4B is ubiquitously expressed in young adult tissues with highest abundance in the cerebrum, cerebellum and skeletal muscles (Kaneko et al., 2003). *Ube4b* null mice die prematurely *in utero* with markedly increased apoptosis in the developing heart (Kaneko-Oshikawa et al., 2005). *Ube4b* heterozygous mice progressively develop neurological symptoms, such as locomotion defects and degeneration of Purkinje cells around 1 year of age. These results indicate that UBE4B may be important for development and maintenance of the brain.

Here, we provide genetic and biochemical evidence demonstrating the indispensable function of UBE4B in brain development. *Ube4b* mRNA is abundant in surrounding lateral ventricles at embryonic and neonatal stages. Loss of UBE4B in the brain causes elevation of a newly discovered substrate, KLHL22, and subsequent hyperactivation of mTOR. This results in severe neurogenesis defects and early lethality associated with spontaneous seizures in newborn mutant animals. Prenatal treatment with the mTOR inhibitor rapamycin successfully rescued premature death and defective neurogenesis caused by *Ube4b* deletion. Our findings have unveiled a crucial physiological function of the highly conserved UBE4B E3/E4 ligase in brain development and provide mechanistic insights into neurodevelopmental disorders associated with aberrant mTOR signaling.

## RESULTS

### Deletion of UBE4B in the CNS results in defective neurogenesis and epilepsy

**Fig. 1.**
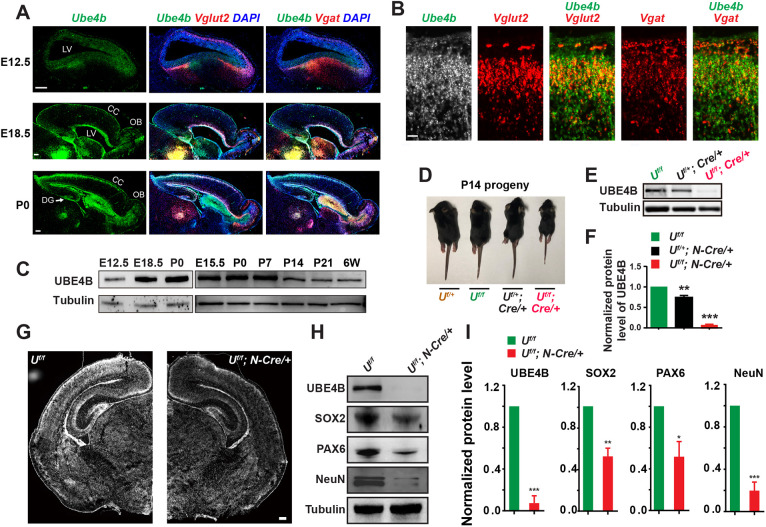
**Deletion of UBE4B in the brain results in loss of NPCs and neurons.** (A) Multiplexed fluorescent RNAScope labeling of UBE4B, VGLUT2 and VGAT in the brain of C56BL/6 mice at E12.5, E18.5 and P0. VGLUT2 and VGAT probes label excitatory and inhibitory neurons, respectively. Cell nuclei were labeled by DAPI. Brain slices were prepared in the sagittal plane. Representative images were selected from three or four samples from each age group. CC, cerebral cortex; DG, dentate gyrus; LV, lateral ventricle; OB, olfactory bulb. Scale bars: 200 μm. (B) Enlarged images of the S1 cerebral cortex region from the P0 brain in A. Scale bar: 20 μm. (C) Immunoblotting of endogenous UBE4B in embryonic and postnatal mouse brains. Left: Whole-brain lysates from E12.5, E18.5 and P0 animals. Right: Whole-brain lysates from E15.5, P0, P7, P14, P21 and 6-week-old (6W) animals. An equal amount of whole brain lysates from C56BL/6 mice were loaded for each experiment. γ-Tubulin was used as the loading control. (D) Representative image of P14 littermates delivered from *Ube4b^f/f^* and *Ube4b^f/+^; Nestin-Cre/+* crossing. Note that the CKO animal was obviously smaller in size than littermate controls. (E) Immunoblotting of UBE4B in whole brains from *Ube4b^f/f^*, heterozygous and homozygous CKO littermates. Representative results from three litters are shown. γ-Tubulin was used as the loading control. (F) Quantification of UBE4B expression in *Ube4b^f/f^*, heterozygous and homozygous CKO littermates in whole brains. Three independent immunoblotting experiments were quantified. Data represent mean±s.e.m. **P<0.01, ***P<0.001 (relative to normalized UBE4B level in *Ube4b^f/f^*; one-way ANOVA). (G) DAPI nuclear counterstaining of sagittal brain sections from P0 *Ube4b^f/f^* (left) and *Ube4b^f/f^; Nestin-Cre/+* (right) littermates. Scale bar: 100 μm. (H) Immunoblotting of endogenous UBE4B, SOX2, PAX6 and NeuN from equal amounts of P0 brain lysates. SOX2 and PAX6 are NPC markers. NeuN is a marker of mature neurons. γ-Tubulin was used as the loading control. (I) Quantification of protein levels of UBE4B, SOX2, PAX6 and NEUN in P0 brains. Whole-brain lysates of three pairs of *Ube4b^f/f^* and *Ube4b^f/f^; Nestin-Cre/+* littermates were immunoblotted and quantified. Data represent mean±s.e.m. *P<0.05, **P<0.01, ***P<0.001 (compared with normalized protein levels in *Ube4b^f/f^* control animals; unpaired, two-tailed Student's *t*-test).

In the mouse brain, *Ube4b* mRNA was expressed during embryonic and neonatal stages and was particularly abundant in regions surrounding the lateral ventricles (Fig. 1A). Multiplexed labeling of *Ube4b* and *Vglut2* (*Slc17a6*) or *Vgat* (*Slc32a1*) by RNAScope probes demonstrated that *Ube4b* was transcriptionally expressed in both excitatory and inhibitory neurons in the postnatal day (P) 0 brain (Fig. 1B). Immunoblotting of embryonic and postnatal brains revealed that the protein level of UBE4B was low at embryonic day (E) 12.5, peaked between E15.5 and P7, and gradually declined afterwards (Fig. 1C). To study the function of UBE4B in the developing brain, we generated a mouse strain carrying a floxed allele of *Ube4b* (*Ube4b^f/f^*) and crossed it to *Nestin-cre/+* mice (Fig. S1A). Protein and mRNA expression of UBE4B in the brain was efficiently abolished in homozygous conditional knockout (CKO) mice (Fig. 1E,F; Fig. S1B). We noticed that half of CKO animals were unable to survive beyond the first week after birth. The remaining often suffered from sudden death and very few animals reached adulthood (Fig. S1C). The body weight of surviving CKO animals was significantly lower than that of *Ube4b^f/f^* littermates (Fig. 1D; Fig. S1D). In contrast, the survival rate and body weight of heterozygous animals (*Ube4b^f/+^; Nestin-cre/+*) were normal compared with *Ube4b^f/f^*, suggesting that *Ube4b* is haplosufficient for survival and body growth (Fig. 1E,F; Fig. S1C,D).

To determine the cause of sudden death of *Ube4b Nestin*-CKO, 1-month-old littermates were simultaneously and continuously video-recorded. We discovered tonic-clonic seizure behaviors in the CKO group. Animals that were experiencing seizures displayed tail curling, back hunching, rhythmic twitching and rolling behaviors (Fig. S1F; Movie 1). Although these epileptic episodes lasted only 1-2 min, the affected animals showed poor recovery and often died within one day. Next, we examined the brain anatomy of P0 littermates and noticed a lower cell density in CKO brains than that in the control group (Fig. 1G). The expression levels of the neural progenitor cell (NPC) markers SOX2 and PAX6 and the neuronal marker NeuN (RBFOX3) were all significantly reduced in CKO brains (Fig. 1H,I), to which lethal seizures might be attributed. In addition, dendritic trees in the cerebral cortex showed mild disorganization (Fig. S1E). These observations suggest that UBE4B is important for neurogenesis in the brain.

### Deletion of UBE4B leads to accumulation of KLHL22

**Fig. 2.**
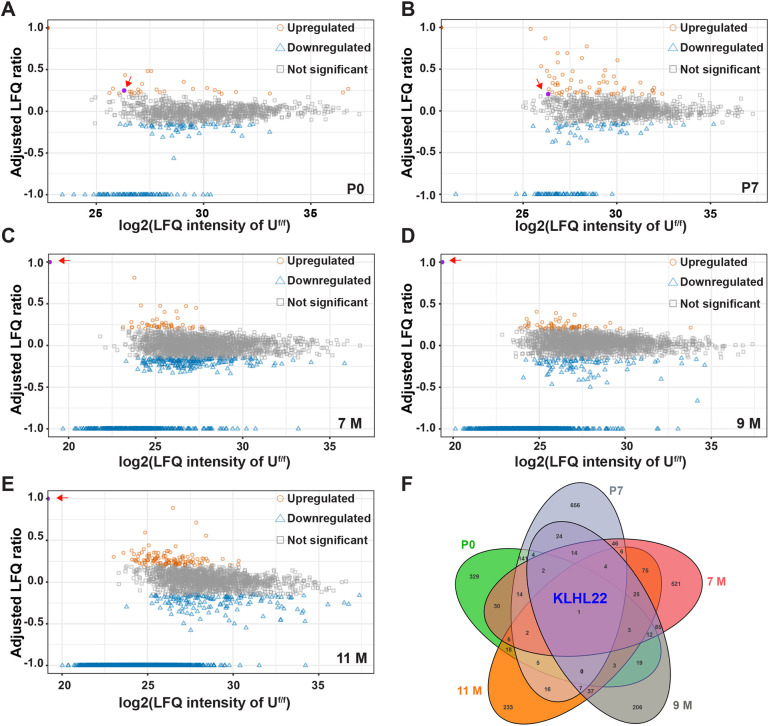
**Deletion of UBE4B causes upregulation of KLHL22 in neonatal and mature brains as revealed by proteomic analyses.** (A,B) Mass spectrometry analysis of postnatal whole brain proteomes following embryonic deletion of UBE4B in NSCs. The *x*-axis of the scatter plots represents log2-transformed label-free quantification (LFQ) intensities of each protein in *Ube4b^f/f^*. The *y*-axis represents adjusted ratio of LFQ intensities between *Ube4b^f/f^; Nestin-Cre/+* versus *Ube4b^f/f^*. Three pairs of littermates at P0 (A) and P7 (B) were analyzed, respectively. Commonly upregulated proteins in *Nestin-*CKO brains from both P0 and P7 are represented by orange circles and commonly downregulated genes are represented by blue triangles. Red arrows point to KLHL22, represented by a purple circle. Details of data analyses are described in Materials and Methods. (C-E) Mass spectrometry analysis of forebrain proteomes from mature adults following postnatal deletion of UBE4B. The *x*-axis of the scatter plots represents log2-transformed LFQ intensities of each protein in *Ube4b^f/f^*. The *y*-axis represents adjusted ratio of LFQ intensities between *Ube4b^f/f^; Camk2a-Cre/+* and *Ube4b^f/f^*. Three pairs of littermates at 7 months (C), 9 months (D) and 11 months (E) of age were analyzed, respectively. Commonly upregulated proteins in *Camk2a-*CKO animals from all three age groups are represented by orange circles and commonly downregulated genes are represented by blue triangles. Red arrows point to KLHL22, represented by a purple circle. (F) Venn diagram showing that KLHL22 is the sole protein upregulated upon UBE4B knockout in all five age groups.

Only a handful of UBE4B substrates have been identified, including the transcription factor p53 (TRP53) (Antoniou et al., 2019). Overexpression of p53 can cause abnormal NSC proliferation and neuronal differentiation (Xiong et al., 2020), which might explain *Ube4b* CKO phenotypes. However, no significant changes of p53 expression were found in *Ube4b* CKO brains (Fig. S2A,B). To evaluate the effects of UBE4B deletion in an unbiased manner and to identify new substrates of UBE4B, we analyzed the brain proteomes of *Ube4b^f/f^* and *Nestin*-CKO littermates at P0 and P7 (Fig. 2A,B) and identified a subset of proteins commonly upregulated or downregulated in UBE4B CKO samples (Fig. 2A,B; Fig. S2C-F).

In addition, we also crossed *Ube4b^f/f^* and *Camk2a-cre* strains to generate *Ube4b Camk2a*-CKO. These mice showed normal body growth and survived to adulthood, suggesting that postnatal deletion of UBE4B in excitatory neurons in the forebrain can avoid neonatal and juvenile lethality. We analyzed the brain proteomes of *Ube4b^f/f^* and *Camk2a*-CKO animals at 7, 9 and 11 months (Fig. 2C-E) and compared differentially expressed proteins caused by *Ube4b* knockout in young and mature brains. Strikingly, KLHL22, an understudied E3 ligase that degrades DEPDC5 in the mTOR pathway (Chen et al., 2018), turned out to be the sole protein upregulated in CKO animals from all five age groups (Fig. 2F; Table S2). These results suggest that KLHL22 may be a substrate of UBE4B in the brain.

### UBE4B promotes ubiquitylation and degradation of KLHL22

**Fig. 3.**
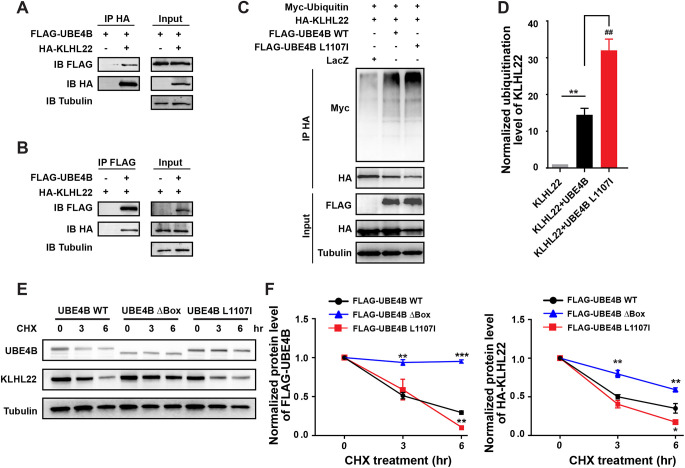
**UBE4B promotes ubiquitylation and degradation of KLHL22.** (A,B) Reciprocal co-IP of FLAG-UBE4B and HA-KLHL22 in HEK293T cells. Cells were treated with the proteasome inhibitor bortezomib (5 µM) for 4 h and lysed for immunoprecipitation with either anti-HA antibody (A) or anti-FLAG antibody (B). γ-Tubulin was used as the loading control of inputs. Results are representative of three independent experiments. (C) UBE4B WT or L1107I (hyperactive mutant) were co-transfected with KLHL22 and Myc-ubiquitin into HEK293T cells as indicated. pcDNA6b-lacZ was used as control. Cells were pre-treated with bortezomib for 4 h before collection for immunoprecipitation with anti-HA antibody. Polyubiquitylation signals of KLHL22 were detected by anti-Myc immunoblotting. (D) Quantification of normalized polyubiquitylation levels of HA-KLHL22 in HEK293T cells overexpressing UBE4B WT or L1107I. KLHL22 polyubiquitylation signals detected by anti-Myc antibody were normalized to the total KLHL22 proteins immunoprecipitated by anti-HA antibody. Then this ratio in UBE4B WT or L1107I-expressing cells was normalized to that in cells transfected with the pcDNA6b-lacZ. Three independent immunoblots were analyzed. Data represent mean±s.e.m. **P<0.01, ^##^*P*<0.01 (relative to the indicated controls; unpaired, two-tailed Student's *t*-test). (E) UBE4B-knockout Neuro2A cells were transfected with HA-KLHL22 and FLAG-UBE4B WT, ΔBox or L1107I. Cells were treated with 1 μM cycloheximide (CHX) for 0, 3 or 6 h before collection. Equal amounts of cell lysates were immunoblotted with the indicated antibodies. (F) Normalized protein levels of FLAG-UBE4B and HA-KLHL22 at different time points as shown in E. Three independent experiments were quantified. Data represent mean±s.e.m. *P<0.05, **P<0.01, ***P<0.001 (relative to FLAG-UBE4B WT-expressing cells at each corresponding time point; one-way ANOVA).

The conserved U-box domain of UBE4B mediates its interaction with E2 and thus is important for its E3/E4 function (Fig. S3A-C) (Ohi et al., 2003). We addressed the question of whether KLHL22 is a ubiquitylation substrate of UBE4B with three experiments. First, physical interaction between FLAG-UBE4B and HA-KLHL22 was confirmed by reciprocal co-immunoprecipitation (co-IP) assays in HEK293T cells following treatment with the proteasome inhibitor bortezomib (Fig. 3A,B). We noticed that both KLHL22 and UBE4B showed relatively diffused cytosolic and nuclear distribution in cells (Fig. S3D). Second, overexpression of both wild-type UBE4B (UBE4B WT) and a hyperactive mutant (L1107I) greatly enhanced KLHL22 polyubiquitylation in cells (Fig. 3C,D). The L1107I mutation enhances ubiquitin transfer from E2 to UBE4B substrates (Starita et al., 2013). Third, when re-introduced into *Ube4b* knockout Neuro2A cells (Fig. S3E), both UBE4B WT and L1107I promoted KLHL22 degradation, whereas the enzymatically dead mutant (UBE4B Δbox) showed the opposite effect and stabilized KLHL22 (Fig. 3E,F). Furthermore, we noticed that UBE4B Δbox also blocked its own degradation, which is consistent with previous knowledge that the U-box domain of UBE4B also mediates its auto-ubiquitylation (Starita et al., 2013). These findings together indicate that KLHL22 is a new substrate of UBE4B.

### UBE4B negatively regulates activation of the mTOR pathway through degradation of KLHL22

In cancer cells and in aging *C. elegans*, KLHL22 promotes mTORC1 activation by degrading DEPDC5 and releasing inhibition of GATOR1 on mTORC1 (Fig. S4A) (Chen et al., 2018). Thus, we decided to investigate whether the effects of UBE4B deletion in brain development may be mediated by KLHL22 and mTOR. We first characterized the spatial and temporal expression of KLHL22 and components of the mTOR pathway in rodent brains. Similar to UBE4B, KLHL22 and mTOR were expressed throughout embryonic and postnatal stages in mouse brain (Fig. S4B). We also checked the expression of UBE4B, KLHL22 and mTOR in different brains regions of P7 rats (Fig. S4C). A significant difference in UBE4B levels was found between the olfactory bulb and cerebellum, with UBE4B being two-fold higher in the latter (Fig. S4D1). Interestingly, an inverse correlation between the amounts of UBE4B and phosphorylated S6K, a common indicator of mTORC1 activity, was found in these two regions.

**Fig. 4.**
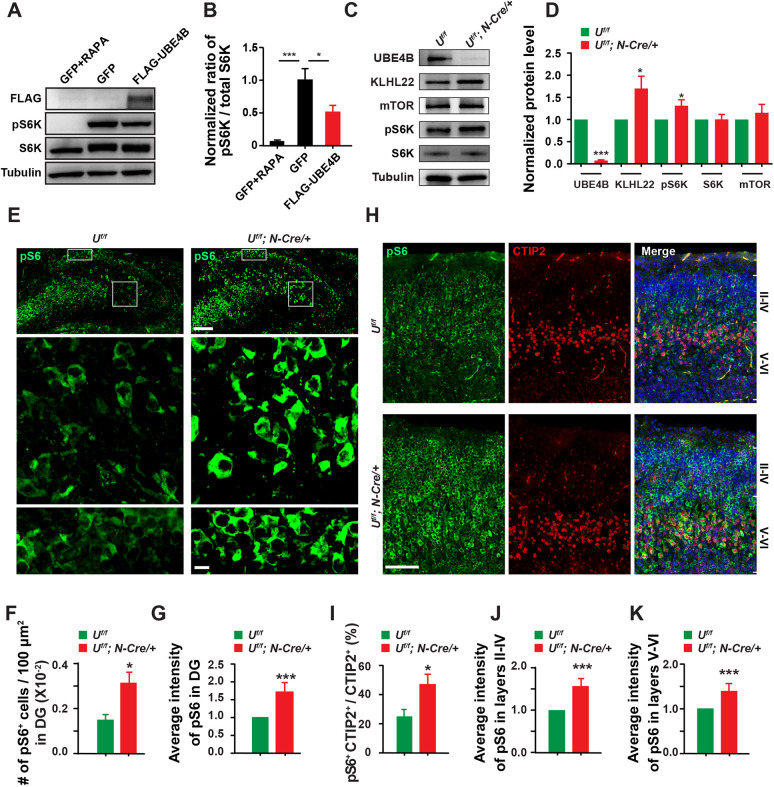
**UBE4B suppresses phosphorylation of S6K and S6 in cultured cells and in the brain.** (A) Overexpression of UBE4B inhibits phosphorylation of endogenous S6 kinase (S6K) in HEK293T cells. HEK293T cells were transfected with GFP or FLAG-UBE4B. Rapamycin (RAPA; 1 µM, 4 h) was applied to inhibit mTOR activity in GFP-expressing cells, which served as a negative control. Cells were collected and blotted with the indicated antibodies. (B) Quantification of the phosphorylated S6K (pS6K) levels shown in A. The ratio of pS6K over total S6K signals in GFP- or FLAG-UBE4B-transfected cells was normalized to that in rapamycin-treated cells. Three independent repeats were analyzed. Data represent mean±s.e.m. *P<0.05, ***P<0.001 (relative to the indicated controls; one-way ANOVA). (C) Accumulation of KLHL22 and enhanced phosphorylation of S6K in the *Ube4b Nestin*-CKO brain. An equal amount of P0 brain lysates were immunoblotted for UBE4B, KLHL22, mTOR, pS6K, S6K and tubulin antibodies. (D) Quantification of the indicated proteins in four pairs of P0 *Ube4b^f/f^* and *Nestin*-CKO littermates. Data represent mean±s.e.m. *P<0.05, ***P<0.001 (relative to the normalized protein levels in *Ube4b^f/^* mice; unpaired, two-tailed Student's *t*-test). (E) Immunohistochemical detection of ribosomal protein S6 phosphorylation (pS6, Ser240/244) in the DG of P0 *Ube4b Nestin*-CKO mice. Boxed regions in the GCL and the hilus are magnified below. Scale bars: 200 μm (top); 20 μm (bottom). (F) Increased density of pS6^+^ cells in the DG of *Ube4b Nestin*-CKO mice. Brain slices from four pairs of P0 *Ube4b^f/f^* and CKO animals were counted and analyzed. (G) Increased pS6 intensity in pS6^+^ cells in the CKO DG. The average intensity of pS6 in pS6^+^ cells from four pairs of P0 *Ube4b^f/f^* and CKO littermates (*n*=221 and 256, respectively) was quantified. (H) Elevated phosphorylation of S6 in the S1 cortex in CKO animals at P0. Neurons in layer V were labeled by CTIP2 and all nuclei were stained by DAPI. The boundary of layers II-IV and layers V-VI was grossly defined based on CTIP2 and DAPI staining. Note that the distribution range of pS6^+^ cells was broader in CKO animals. Scale bar: 200 μm. (I) Increased ratio of pS6^+^ CTIP2^+^ cells in CTIP2^+^ cells in the S1 cortex. Brain slices from three pairs of P0 *Ube4b^f/f^* and CKO littermates were counted and analyzed. pS6 images from both groups were set to the same threshold. The ratio of pS6^+^ CTIP2^+^ cells in CTIP2^+^ cells was calculated as the number of CTIP2^+^ cells with pS6 signals above the threshold versus the total number of CTIP2^+^ cells. (J,K) Quantification of pS6 intensity in pS6^+^ cells in the layers II-IV (J) and V-VI (K). Cells from three pairs of P0 *Ube4b^f/f^* and CKO littermates were quantified (*n*=147 and 124, respectively, in J; *n*=178 and 165, respectively, in K). Data represent mean±s.e.m. *P<0.05, ***P<0.001 (relative to *Ube4b^f/f^*; unpaired, two-tailed Student's *t*-test).

In line with the above findings, UBE4B overexpression significantly suppressed phosphorylation of endogenous S6K in HEK293T cells (Fig. 4A,B). In *Ube4b Nestin*-CKO animals, the whole-brain expression of both KLHL22 and phosphorylated S6K was upregulated (Fig. 4C,D), whereas the total levels of S6K and mTOR were unchanged compared with those in wild-type animals. Immunohistochemistry of brain slices showed that deletion of UBE4B caused an induction of S6 phosphorylation in the dentate gyrus (DG) and cerebral cortex of P0 brains (Fig. 4E-H). The density of phospho-S6 (pS6)^+^ cells and the average intensity of pS6 signals were both increased in the granular cell layer (GCL) and the hilus regions (Fig. 4F,G). In CKO brains, the percentage of pS6^+^ neurons labeled by the granule cell (GC) marker CTIP2 (BCL11B) was doubled, and pS6^+^ neurons showed broader distribution in S1 cortex (Fig. 4H,I). The average intensity of pS6 signals in the layers II-IV and V-VI was also elevated (Fig. 4J,K). The soma size of neurons in the DG was increased in the CKO brains (Fig. S5A,B). Increased pS6 phosphorylation and enlarged cell soma, typical consequences of mTOR hyperactivation, strongly indicate that UBE4B negatively regulates mTOR signaling *in vivo*.

**Fig. 5.**
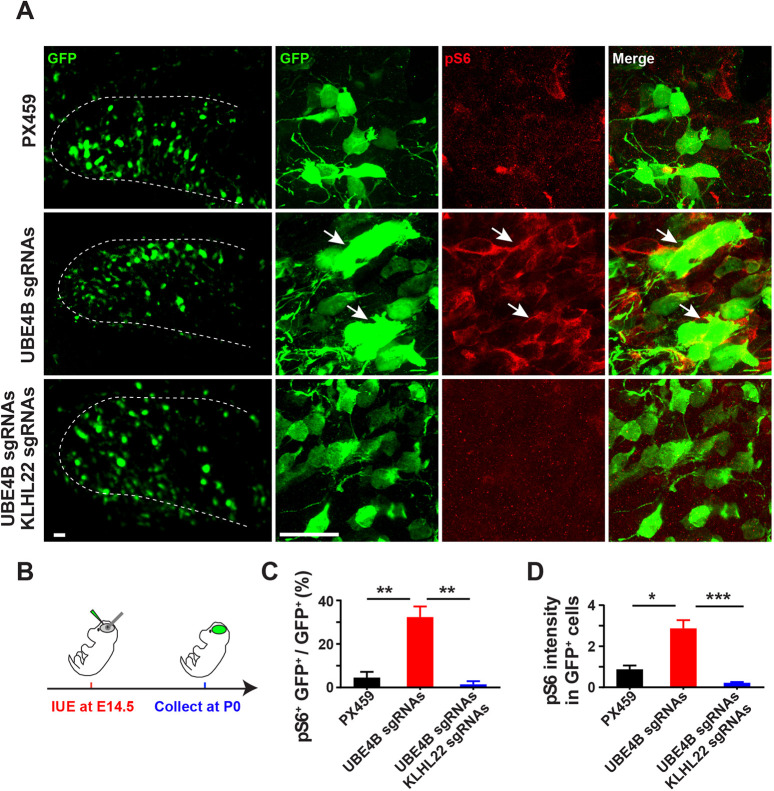
**Acute deletion of UBE4B in the forebrain induces KLHL22-dependent mTOR hyperactivation.** (A,B) *Ube4b* sgRNAs (in the PX459 vector) were delivered with or without *Klhl22* sgRNAs into the hippocampal region of E14.5 embryos by *in utero* electroporation (as illustrated in B). pCIG2-GFP was co-delivered to mark electroporated cells. Brains were collected at birth and pS6 signals were examined. Images of GFP^+^ cells in the DG were acquired at 10× (left) and 100× magnification (right), respectively. Arrows indicate cells with elevated pS6 signals. Dashed line delineates the DG region. Scale bars: 50 μm. (C,D) Ratio of pS6^+^ cells (C) and average intensity of pS6 signals (D) in GFP-expressing cells in electroporated DGs at P0. Three animals for each experimental group were quantified. Data represent mean±s.e.m. *P<0.05, **P<0.01, ***P<0.001 (relative to indicated controls; one-way ANOVA).

To determine whether mTOR activation by UBE4B deletion is dependent on KLHL22, we generated sgRNAs to eliminate KLHL22 in UBE4B-deleted cells. First, UBE4B was acutely knocked out by injecting *hSyn1-Cre*-carrying AAV virus into the lateral ventricles of P0 *Ube4b^f/f^; Cas9/Cas9* mice (Kim et al., 2014; Hana et al., 2021). Downregulation of UBE4B and upregulation of KLHL22 in the infected cortical regions were confirmed by immunoblotting (Fig. S5D). Next, co-injection of AAV viruses expressing *hSyn1-Cre* and validated KLHL22 sgRNAs (Fig. S5C) was able to suppress upregulation of KLHL22 (Fig. S5D). Then, we locally abolished UBE4B expression in the DG by *in utero* electroporation of UBE4B sgRNA plasmids at E14.5 (Fig. 5A,B). Electroporated brains were collected at birth for examination of mTOR activity. Similar to what was observed in *Ube4b* CKO mice, a significantly higher ratio of cells expressing UBE4B sgRNAs were pS6 positive (Fig. 5C). The average intensity of pS6 signals in these cells was also increased (Fig. 5D). Moreover, co-expression of KLHL22 sgRNAs with UBE4B sgRNAs was able to suppress enhancement of S6 phosphorylation caused by UBE4B deletion (Fig. 5C,D). These data together indicate that UBE4B negatively modulates mTOR activity by regulating KLHL22 expression.

### UBE4B regulates neurogenesis through mTOR in the developing brain

**Fig. 6.**
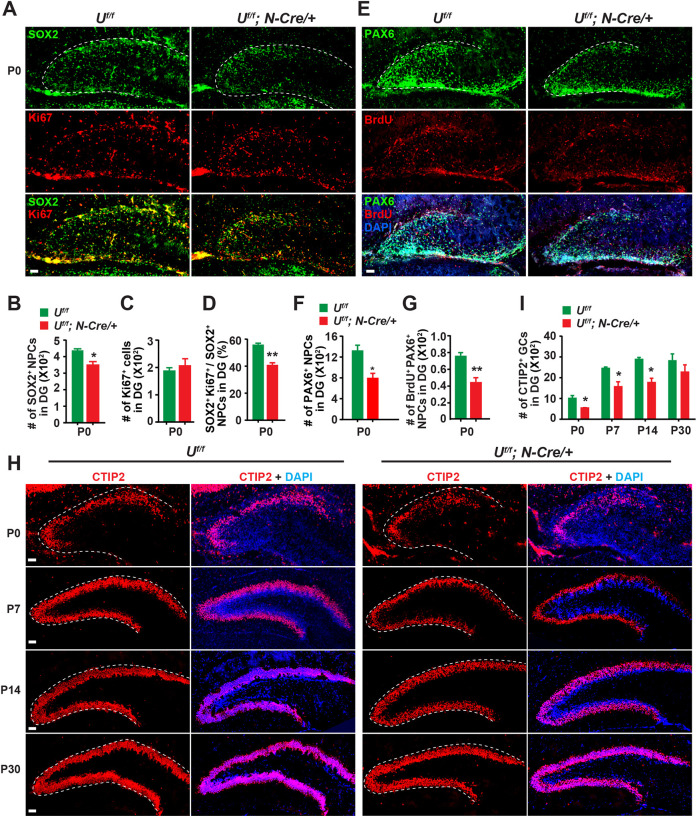
**Deletion of UBE4B in the brain impairs proliferation and differentiation of NPCs.** (A-D) Immunohistochemistry of SOX2 and Ki67 in the DG at P0 (A). Scale bar: 50 μm. Three pairs of P0 *Ube4b^f/f^* and *Ube4b Nestin*-CKO littermates were analyzed for the number of SOX2^+^ NPCs (B), the number of Ki67^+^ proliferating cells (C) and the percentage of proliferating NPCs (SOX2^+^ Ki67^+^) (D) in the DG. Data represent mean±s.e.m. *P<0.05, **P<0.01 (relative to *Ube4b^f/f^*; unpaired, two-tailed Student's *t*-test). (E-G) Reduction of proliferating and total PAX6^+^ NPCs in the DG of *Ube4b Nestin*-CKO. Scale bar: 50 μm. A single pulse of BrdU was injected into pregnant dams at E19.5 to label proliferating cells. Injected mice were sacrificed 12 h later. Genotype-confirmed *Ube4b^f/f^* and *Ube4b Nestin*-CKO pups were processed for BrdU and PAX6 staining. BrdU^+^ PAX6^+^ cells were considered proliferating NPCs. Brain slices from three pairs of *Ube4b^f/f^* and CKO pups were counted and analyzed (F,G). Data represent mean±s.e.m. *P<0.05, **P<0.01 (relative to *Ube4b^f/f^*; unpaired, two-tailed Student's *t*-test). (H,I) Immunohistochemistry (H) and quantification (I) of CTIP2^+^ GCs in the DG at the indicated postnatal stages. Scale bars: 50 μm. *n*=3, 3, 4 and 3 pairs of *Ube4b^f/f^* and CKO littermates for P0, P7, P14 and P30, respectively. Data represent mean±s.e.m. *P<0.05 (relative to *Ube4b^f/f^* at the same age; unpaired, two-tailed Student's *t*-test).

Hyperactivation of mTOR is often observed in neurodevelopmental mTORopathies and associated with characteristic defects, such as abnormal architecture of the cerebral cortex and hippocampus (Meikle et al., 2007; Magri et al., 2011; Zhou et al., 2011; Carson et al., 2012). Immunoblotting of whole-brain lysates demonstrated that UBE4B deletion caused a significant decrease of SOX2, PAX6 and NeuN expression, suggesting impaired neurogenesis (Fig. 1H,I). In the DG of rodents, most GCs are born postnatally, a substantial fraction of which appear during the first week (Eriksson et al., 1998; Li and Pleasure, 2005; Bond et al., 2020). Perturbation of DG neurogenesis is one of the most common causes for epilepsy and learning and memory deficits (Scharfman, 2019). Thus, we examined markers of the GC lineage in the DG to examine further the consequences of *Ube4b* deletion. The transcription factors SOX2 and PAX6 mark NPCs, including NSCs and type 1 intermediate progenitor cells (IPCs), in the DG (Sugiyama et al., 2013). At the P0 stage, SOX2^+^ NPCs were found in areas surrounding the GCL and in the hilus (Fig. 6A). Consistent with the immunoblotting result, the number of SOX2^+^ NPCs was significantly reduced in the DG of *Ube4b Nestin*-CKO animals (Fig. 6A,B). A similar phenomenon was also observed in the cerebral cortex and midbrain regions (Fig. S6A,B). We also quantified proliferating NPCs, i.e. cells that were co-labeled by SOX2 and the proliferation marker Ki67 (MKI67), in the DG. A decline in the ratio of proliferating NPCs in the NPC pool was observed in CKO animals (Fig. 6C,D).

To confirm these results, pregnant dams were injected with a single pulse of bromodeoxyuridine (BrdU) at E19.5 and pups were collected for BrdU and PAX6 staining 12 h later (Magri et al., 2011; Cloëtta et al., 2013). Compared with *Ube4b^f/f^* littermates, the numbers of both PAX6^+^ NPCS and PAX6^+^ BrdU^+^ proliferating NPCs were decreased in homozygous but not in heterozygous CKO animals (Fig. 6E-G; Fig. S6C,D). In addition, the GFAP-marked radial glial scaffold was also noticeably disorganized (Fig. S6G,H). Tbr2^+^ (EOMES) type 2 IPCs were greatly reduced in the DG (Fig. S6E,F), suggesting that proliferation and differentiation of NPCs were impaired by UBE4B deletion. Consistent with this, the numbers of CTIP2^+^ mature GCs in neonatal and postnatal CKO DGs were also significantly lower than those in control brains until P30 (Fig. 6H,I). In contrast, no changes in CTIP2^+^ GCs were detected in heterozygous CKOs (Fig. S6C,D), suggesting that one allele of UBE4B is sufficient to maintain postnatal neurogenesis. We also examined PAX6^+^ NPCs and PROX1^+^ GCs at E14.5 and E18.5. As expected, the numbers of NPCs and GCs in embryonic DGs were much lower than those in postnatal DGs (Fig. S6I-L). However, no difference was found between control and CKO littermates (Fig. S6J,L). Thus, we conclude that UBE4B is of particular importance during the neonatal burst of neurogenesis within the first few weeks.

**Fig. 7.**
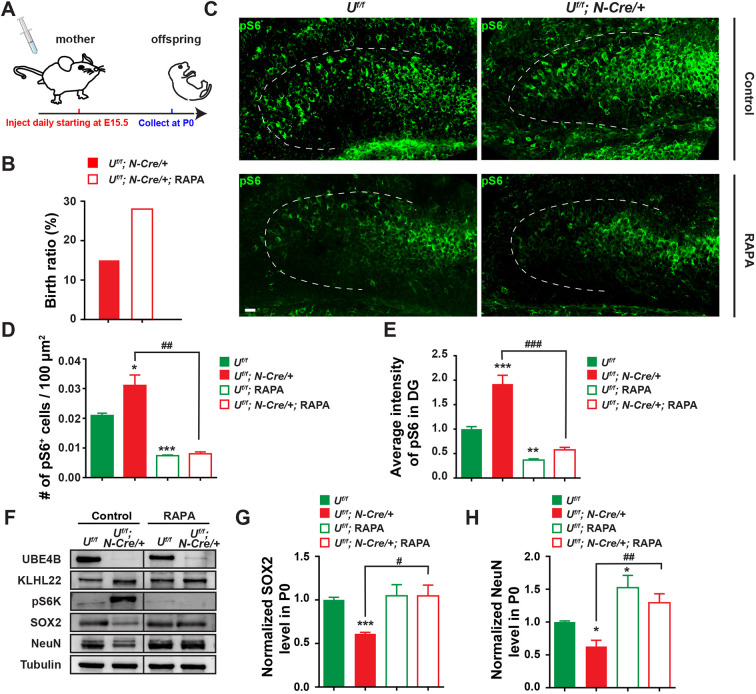
**mTOR hyperactivation and neurogenesis defects caused by UBE4B deletion are rescued by prenatal treatment of rapamycin.** (A) Schematic of the rapamycin injection strategy. Pregnant *Ube4b^f/f^* mice mated with *Ube4b^f/+^; Nestin-Cre/+* were treated daily by intraperitoneal injection of rapamycin (RAPA) (3 mg/kg of body weight) or control vehicle (corn oil) starting from E15.5. P0 pups were collected immediately at birth for immunohistochemistry and biochemistry. (B) The birth ratio of *Ube4b* CKO animals is increased by rapamycin treatment. After prenatal injection of rapamycin, the total numbers of *Ube4b^f/+^*, *Ube4b^f/f^*, heterozygous and homozygous CKO animals at birth from all injected females were 10, 11, 9 and 12, respectively. (C) Immunohistochemistry of pS6 in the DGs of newborn *Ube4b^f/f^* and homozygous CKO animals born by mothers injected with vehicle control or rapamycin. Scale bar: 50 μm. (D,E) Quantification of the density of pS6^+^ cells (D) and the average intensity of pS6 signals (E) in the DG of newborn *Ube4b^f/f^* and CKO animals prenatally treated with vehicle control or rapamycin. Brain sections from three pairs of *Ube4b^f/f^* and CKO pups were analyzed for the control and rapamycin groups, respectively. Data represent mean±s.e.m. *P<0.05, **P<0.01, ***P<0.001 (relative to *Ube4b^f/f^* treated with vehicle control); ^##^*P*<0.01, ^###^*P*<0.001 (relative to CKO treated with vehicle control) (one-way ANOVA). (F) The effects of prenatal rapamycin injection on protein levels in newborn *Ube4b^f/f^* and *Ube4b^f/f^; Nestin-Cre/+* animals. After rapamycin treatment of pregnant females (A), the forebrain tissues of newborn pups were collected and immunoblotted for indicated antibodies. Phosphorylation of S6K indicates mTOR activity. SOX2 and NeuN are markers for NPCs and mature neurons, respectively. (G,H) Normalized expression levels of SOX2 (G) and NeuN (H) in the forebrain from newborn *Ube4b^f/f^* and *Ube4b^f/f^; Nestin-Cre/+* animals prenatally treated with vehicle control or rapamycin. *n*=3 per group. Data represent mean±s.e.m. *P<0.05, ***P<0.001 (relative to *Ube4b^f/f^* treated with vehicle control); ^#^*P*<0.05, ^##^*P*<0.01 (relative to CKO treated with vehicle control) (one-way ANOVA).

Next, we investigated whether the effects of UBE4B deletion in the brain were due to mTOR hyperactivation. To address this question, we inhibited the mTOR pathway by daily intraperitoneal injection of rapamycin into pregnant mice starting from E15.5 (Fig. 7A). Consistent with our prediction, premature death of *Ube4b Nestin*-CKO progeny was rescued and the birth rate was restored from 14.0% to a level close to the Mendelian ratio (28.5%) by prenatal rapamycin treatment, indicating that mTOR hyperactivation was the cause of death (Fig. 7B). Aafter rapamycin treatment, the density of pS6^+^ cells and the average intensity of pS6 signals in the CKO DGs were both greatly suppressed (Fig. 7C-E). Strong suppression of S6 phosphorylation was also observed in the cortical cortex of CKO animals after drug treatment (Fig. S7A-D). Reduced pS6 signals were also, unsurprisingly, observed in control animals compared with those injected with the vehicle control (Fig. 7C-E).

We also investigated whether mTOR inhibition could reverse defective neurogenesis in *Ube4b Nestin*-CKO animals. Whole brains of offspring from rapamycin-treated pregnant females were collected at birth and immunoblotted for SOX2 and NeuN. As predicted, rapamycin treatment abolished elevated pS6K signals in CKO animals but did not alter upregulation of KLHL22 (Fig. 7F), suggesting that KLHL22 functions upstream of mTOR. Importantly, the expression of SOX2 and NeuN in CKO brain was fully restored to a normal level by mTOR inhibition (Fig. 7G,H). The number of PAX6^+^ NPCs was also recovered in the DG region (Fig. S7E-G). These findings together clearly demonstrate that fine-tuning of mTOR activity by UBE4B and KLHL22 is essential for neonatal neurogenesis in the developing mouse brain.

## DISCUSSION

In this study, we report that the E3/E4 ubiquitin ligase UBE4B negatively controls mTOR signaling during neonatal brain development. Mechanistically, UBE4B promotes the polyubiquitylation and degradation of KLHL22, preserving the GATOR1 component DEPDC5. Chronic and acute knockout of UBE4B causes upregulation of KLHL22 and release of GATOR1 inhibition on mTOR, which eventually leads to mTOR hyperactivation and elevated S6 phosphorylation. In mutant animals, aberrant mTOR signaling is accompanied by a reduction of the NPC pool, and neurogenesis defects, seizures and premature death. Inhibition of mTOR can suppress cellular changes, neurogenesis defects and premature death induced by UBE4B deletion, suggesting that mTOR hyperactivation is the major cause of these phenotypes. In summary, the UBE4B-KLHL22 E3 cascade maintains a healthy level of mTOR activity and ensures proper self-renewal and differentiation of NPCs (Fig. S7H). These findings define UBE4B as an important regulator of mTOR signaling.

The *UBE4B* gene has been found to be amplified at a high frequency in various types of brain tumors (Wu et al., 2011; Antoniou et al., 2019). UBE4B promotes tumorigenesis by degrading the tumor suppressor p53. In neuroblastoma cells, UBE4B participates in lysosomal degradation of EGFR (Zage et al., 2013; Sirisaengtaksin et al., 2014; Memarzadeh et al., 2019). In SCA3 and AD animal models, UBE4B facilitates ubiquitylation and degradation of ataxin 3 and APP, respectively (Matsumoto et al., 2004; Gireud-Goss et al., 2020). Here, we find that KLHL22 is a new ubiquitylation substrate of UBE4B. The UBE4B-KLHL22-DEPDC5 regulatory cascade keeps the activity of mTOR at a level that is optimal for self-renewal and differentiation of NPCs. Deletion of UBE4B in the nervous system caused phenotypes resembling characteristics of mTORopathies reported in mutant mouse models of other mTOR regulators (Crino, 2015; Liu and Sabatini, 2020; Parenti et al., 2020). However, the brain size and the thickness of cerebral cortex appeared normal in *Ube4b* knockout mice, with no signs of megalencephaly, which is often observed in animal models of mTOR hyperactivation. One possible explanation is that the reduction in cell numbers offset the enlarged soma size, so that the brain volume remained largely unchanged in *Ube4b* CKO animals. Although the level of p53 is unaltered in CKO brains at birth, it remains to be determined whether dysregulation of UBE4B substrates other than KLHL22 might have contributed to the *Ube4b* knockout phenotypes.

In recent years, a few new regulators of mTOR signaling have been identified from human pathological studies and genetic studies in model organisms. For example, the S-adenosylmethionine (SAM) sensor SAMTOR binds to GATOR1 and inhibits mTOR in response to leucine, arginine and SAM (Gu et al., 2017). The KLHL22-CUL3 E3 ligase acts as a positive regulator of mTOR in cancer cells and in *C. elegans* (Chen et al., 2018). However, it is unknown whether KLHL22 is also involved in mTOR regulation in the nervous system. Here, we report that UBE4B promotes ubiquitylation and degradation of KLHL22 and eventually suppresses mTOR signaling in the brain. UBE4B is particularly abundant in regions enriched with NPCs during embryonic and neonatal stages, with a declining tendency in older pups (Fig. 1A-C). In P7 rat brains, UBE4B shows varied expression in different brain regions with highest abundance in the cerebellum, where the level of pS6 is lowest (Fig. S4C,D). A reasonable prediction is that dynamic expression of UBE4B and its substrates could refine the regulation network of mTOR to suit the spatiotemporal requirements of neurogenesis and brain growth. It will be important to investigate further whether UBE4B is regulated in response to intrinsic and extrinsic factors and how such regulation relates to its function in mTOR signaling.

In rodents, the net number of neurons is approximately doubled in the cerebral cortex and hippocampus during the first postnatal week, followed by massive gliogenesis and neuronal death in the next few weeks (Altman and Bayer, 1990; Li and Pleasure, 2005; Bandeira et al., 2009). Sustained neurogenesis depends on maintenance of the NPC pool, which is determined by several processes, including proliferation, differentiation and survival at any given moment. Deletion of UBE4B in the nervous system causes severely reduced NPCs, IPCs and GCs in the neonatal DG. The absence of UBE4B may impair neurogenesis at several steps: (1) the cell cycle progression of NPCs; (2) the differentiation of NPCs into IPCs and GCs; or (3) the survival of NPCs and GCs. Further investigations will be needed to determine at which step(s) UBE4B participates. A close examination of events downstream of mTOR, including cell cycle progression, gene transcription, protein synthesis, autophagy and apoptosis, in UBE4B-deleted cells and animals will provide important mechanistic clues to this question.

In summary, our findings demonstrate that UBE4B and its substrate KLHL22 are pivotal for brain development by regulating the mTOR signaling. Because the upregulation of KLHL22 by UBE4B deletion is also detected in mice from 7 to 11 months old (Fig. 2), future studies can focus on whether UBE4B and KLHL22 are important for the function and maintenance of mature and aging brains. In addition, although many components of the mTOR pathway have been identified, therapeutic studies of mTORopathies and aging-related diseases are still confounded by the metabolic side effects of current mTOR inhibitors. Recent studies have developed activity-enhancing mutations of UBE4B and small ubiquitin variants that specifically act on UBE4B by modulating its binding to E2 (Starita et al., 2013; Gabrielsen et al., 2017). Genetic or vehicle delivery of these protein tools *in vivo* may provide new intervention strategies for mTOR diseases and aging.

## MATERIALS AND METHODS

### Mouse model

All mouse strains were generated and maintained in the C57BL/6 background. The floxed *Ube4b* mouse strain was generated by inserting LoxP sites into intron 2 and intron 4 (Fig. S1A) (Biocytogen). *Ube4b^flox/flox^* homozygous mice were crossed to *Nestin-Cre* (Tronche et al., 1999) and *Camk2a*-Cre (Tsien et al., 1996) lines, respectively. Next, heterozygous knockout mice were crossed to *Ube4b^flox/flox^* to obtain CKO progeny. The *Ube4b^flox/+^; Nestin-Cre/+* strain was maintained by mating *Ube4b^flox/flox^* and *Ube4b^flox/+^; Nestin-Cre/+*. The *Ube4b^flox/flox^; Camk2a-Cre/+* strain was maintained by mating *Ube4b^flox/flox^* and *Ube4b^flox/flox^; Camk2a-Cre/+*. Paired littermates were used for all CKO studies. *Ube4b^flox/flox^* was also crossed to B6;129-Gt(ROSA)26Sor^tm1(CAG-cas9*,EGFP)Fezh^/J to obtain the *Ube4b^flox/flox^*; *Cas9/Cas9* strain. The numbers and body weight of surviving progeny were quantified every week after birth. All research and animal care procedures followed the guidelines of the National Institutes of Health of China.

Mouse strains used in this study are listed in Table S1. Genotyping primers and sizes of PCR products for the *Ube4b floxed* allele were: LoxP-F GCTGCATGTTTTTGAGGTCGGTCAG, LoxP-R GCCTCCCTGAGTTTTTACTTGCTGC (WT=220 bp, floxed=321 bp). Genotyping primers and sizes of PCR products for *Nestin-Cre* and *Camk2a-Cre* alleles were: *Nestin-*Cre-F ATTTGCCTGCATTACCGGTCG, *Nestin-*Cre-R CAGCATTGCTGTCACTTGGTC (320 bp for the *Cre* allele and no band for the wild-type allele).

### DNA constructs and antibodies

*Ube4b* cDNA was a gift from Dr Rachel Klevit (Starita et al., 2013). This cDNA was subcloned into the pcDNA6b-3XFLAG backbone to make pcDNA6b-3XFLAG-UBE4B WT. pcDNA6b-3XFLAG-UBE4B L1107I was made by site-directed mutagenesis. pcDNA6b-3XFLAG-UBE4B ΔBox was made by deleting the last 75 amino acids from UBE4B. pcDNA3.1-HA-KLHL22 and pBOBI-Myc-Ubiquitin were gifts from Dr Ying Liu (Chen et al., 2018). pcDNA6b-lacZ/V5, which does not contain the FLAG epitope, was a gift from Dr Michael Ehlers (formerly Duke University, NC, USA; currently Limelight Bio) and used as a negative control in the indicated experiments (Wang et al., 2008). All plasmids have been verified by sequencing.

Primary antibodies used for immunoblotting were: rabbit anti-UBE4B (Thermo Fisher Scientific, PA5-22023, 1:10,000), rabbit-anti-UBE4B (HUABIO, ET7111-11, 1:5000), mouse anti-HA (BioLegend 901515, 1:1000), rabbit anti-HA (CST C29F4,1:1000), mouse anti-FLAG (Sigma-Aldrich, F1804, 1:5000), mouse anti-Myc (Santa Cruz Biotechnology, sc-40, 1:1000), mouse anti-GFP (Thermo Fisher Scientific, MA5-15256, 1:10,000), mouse anti-γ-tubulin (Sigma-Aldrich, T6557, 1:10,000), mouse anti-β-actin (Proteintech 66009-1-lg, 1:1000), mouse anti-SOX2 (Abcam, ab79351, 1:1000), mouse anti-NeuN (Millipore, MAB377, 1:1000), mouse anti-p53 (Proteintech, 60283-2-lg, 1:1000), rabbit anti-PAX6 (MBL International, PD022, 1:1000), rabbit anti-KLHL22 (Proteintech, 16214-1-AP, 1:1000), mouse anti-mTOR (Proteintech, 66888-1-lg, 1:1000), rabbit anti-S6K (Cell Signaling Technology, 9202S, 1:1000) and rabbit anti-pS6K (Thr389) (Cell Signaling Technology, 9234S, 1:1000). Conjugated secondary antibodies were used at 1:10,000.

Primary antibodies used for immunocytochemistry and immunohistochemistry were: rabbit anti-MAP2 (Proteintech, 17490-1-AP, 1:1000), rabbit anti-NeuN (Proteintech, 26975-1-AP, 1:1000), mouse anti-S6 (Cell Signaling Technology, 2317S, 1:1000), rabbit anti-pS6 (Ser240/244) (Cell Signaling Technology, 5364S, 1:1000), mouse anti-SOX2 (Abcam, ab79351, 1:1000), rabbit anti-Ki67 (Abcam, ab16677, 1:400), mouse anti-BrdU (Millipore, MAB3222, 1:200), rat L1107anti-CTIP2 (Abcam, ab18465, 1:1500), rabbit anti-PROX1 (Millipore, AB5475, 1:1000), rabbit anti-TBR2 (Millipore, AB2283, 1:300), rabbit anti-UBE4B (Abcam, ab126759, 1:200), mouse-anti-GFAP (Proteintech, 60190-1-Ig, 1:1000), mouse anti-HA (BioLegend, 901515, 1:1000). Cell nuclei were stained with DAPI (Sigma-Aldrich, D9542, 1 μg/ml). Fluorophore-conjugated secondary antibodies were used at 1:500-1:1000. Details of all plasmids and antibodies used in this study are listed in Table S1.

### Cas9/CRISPR

Multiple sgRNAs targeting *Ube4b* and *Klhl22*, respectively, were designed using the Zhang Lab Guide Design Resource (https://zlab.bio/guide-design-resources) and subcloned into pSpCas9(BB)-2A-Puro (PX459) and pAAV-CAG backbones. Their editing efficiency was evaluated by SURVEYOR assays and immunoblotting. *Ube4b* sgRNAs tested for this study were: ATCTCTGTCGCGGTCCTTCC, CGAATGTAGCGACGACAACTAGG and TTCAGCATGAGATTGCGGTCTGG. *Klhl22* sgRNAs tested for this study were: CTCATTCCGGTGGTACATAAGGG, ACAGTTGTACATCTCGTCAGTGG, CACAACCGAGGCAGCCGTACAGG and CCTCAGCAGCAATCGCCTGGAGG. To create *Ube4b*-knockout cell lines, Neuro2A cells were transfected with a pair of *Ube4b* sgRNAs and selected with 1 µg/ml puromycin for 4 days. Surviving single-cell clones were expanded and immunoblotted for UBE4B to identify knockout clones.

### Cell culture and confocal imaging

HEK293T cells and Neuro2A cells were grown in DMEM (Thermo Scientific) supplemented with 10% fetal bovine serum (Gibco). For immunocytochemistry, cells were transfected with Lipofectamine 2000 (Thermo Scientific) and then fixed for immunostaining with the indicated antibodies 24 h later as previously described (Wang et al., 2013). Confocal images were acquired using an Andor spinning disk confocal microscope with 60× and 100× (1.4 N.A.) objectives. Images were processed and analyzed using ImageJ software (NIH) and MetaMorph software (Universal Imaging Corporation).

### Biochemistry

Homogenization of brain tissues was performed in brain lysis buffer (in mM): 40 HEPES, pH 7.4, 2 EDTA, 2 EGTA, 150 NaCl, 1% Triton X-100) at a ratio of 1:10 (w/v). All buffers used in biochemical experiments were supplemented with protease inhibitors and phosphatase inhibitors (Sigma-Aldrich). Equal amounts of proteins were then denatured in SDS sample buffer, separated by SDS-PAGE, and transferred to 0.45-μm PVDF membrane. Co-IP was performed 24 h after transfection of HEK293T cells with the indicated constructs. Cells were treated with 5 µM bortezomib for 4 h and collected in lysis buffer (in mM): 50 Tris, pH 7.5, 150 NaCl, 0.5 EDTA, 0.5% Triton X-100. To immunoprecipitate FLAG-UBE4B or HA-KLHL22, precleared cell lysates were incubated with 2 µg mouse anti-Flag or rabbit anti-HA antibodies for 2 h and then incubated with anti-mouse or anti-rabbit nanobody agarose beads (KT health, KTSM1341 and KTSM1342) at 4°C for 1 h. Thoroughly washed beads were processed for SDS-PAGE and immunoblotting with mouse anti-HA or rabbit anti-FLAG antibodies.

To examine polyubiquitylation of KLHL22, HEK293T cells transfected with the indicated constructs were treated with 5 µM bortezomib for 4 h and collected in lysis buffer with 1% SDS following previous procedures (Wang et al., 2022). In addition to protease and phosphatase inhibitors, 10-mM N-ethylmaleimide was added in this experiment to inhibit deubiquitinase activity. Cell lysates were then diluted with 9 volumes of lysis buffer and needle-sheared 20 times to break genomic DNA. Precleared lysates were used for immunoprecipitation with rabbit anti-HA antibody and immunoblotting with the indicated antibodies.

To monitor the degradation of HA-KLHL22, *Ube4b* knockout stable cells were transfected with UBE4B WT or mutant constructs, as indicated. Next day, cells were treated with 1 µM cycloheximide to block protein synthesis for 0, 3 and 6 h and then collected for immunoblotting as described above. Chemiluminescence images were acquired with the Bio-Rad ChemiDoc Touch Imaging System and analyzed using Image Lab software. For quantification of all biochemistry experiments, three to four biological repeats were performed and analyzed.

### RNAScope

P0 mouse brains were dissected and immediately fixed in 4% paraformaldehyde at 4°C overnight. Sucrose-dehydrated tissues were embedded in Tissue-Tek O.C.T cryotissue-embedding compound (Sakura). Sagittal sections (15-μm thick) prepared by cryostat sectioning were labeled with fluorescent RNAScope probes recognizing *Ube4b*, *Vgat* and *Vglut2*. The whole procedure followed the User Manual of the RNAScope Multiplex Fluorescent Reagent Kit v2 Assay (ACD, 323120). Images were acquired with the Olympus VS120 virtual slide system (10× and 20× tile scan) and processed with MetaMorph software.

### Immunohistochemistry, BrdU labeling and prenatal rapamycin treatment

Embryonic and neonatal mouse brains were dissected and immediately fixed in 4% paraformaldehyde at 4°C overnight. P7 and older mice were fixed by the transcardial perfusion fixation method. Dehydrated tissues (bathed in 30% sucrose solution for 24-36 h) were then embedded in Tissue-Tek O.C.T cryo-embedding compound (Sakura). Next, 25-μm-thick coronal cryo-sections were permeabilized with blocking solution (0.1% Triton X-100, 10% normal goat serum or normal donkey serum in PBS) for 1 h at room temperature. Sections were then incubated with primary antibodies at 4°C overnight followed by secondary antibodies for 1 h at room temperature. Before mounting, brain slices were counterstained with DAPI. Mounted slices were imaged with an Andor spinning disk confocal microscope with 10× or 60× objective or with the Olympus VS120 virtual slide system (10× and 20× tile scan). Images were analyzed using MetaMorph.

To label proliferating NPCs, a single pulse of BrdU (100 mg/kg body weight) was intraperitoneally injected into pregnant mice at E19.5. The timing of pregnancy was determined by identification of a vaginal plug (E0.5). Mice were sacrificed 12 h post-injection, and brains of pups were fixed for immunohistochemistry with PAX6 and BrdU antibodies. For prenatal treatment with rapamycin, pregnant mice at E15.5 were intraperitoneally injected with rapamycin (3 mg/kg body weight) or corn oil daily until giving birth to litters. In this experiment, rapamycin and corn oil cannot be administrated into the same dam. To reduce genetic variations between litters, a *Ube4b^f/+^; Nestin-cre/+* male was crossed with two or three *Ube4b^f/f^* female siblings and pregnant females were used for injections. Newborn pups were collected immediately for genotyping and brains from *Ube4b^f/f^* and CKO pups were used for analysis. For quantification of NPC and GC numbers, the values were averaged across 9-15 brain sections per animal to cover the rostro and medial levels of the DG. The S1 cortex area above the DG in these brain sections was analyzed as well. For quantification of pS6 signal intensity, the average intensity of pS6 signals from cells across six to nine brain slices in three pairs of control and CKO animals was analyzed. pS6 images from littermates were set to the same threshold. pS6 images from littermates were set to the same threshold. Cells with pS6 signals above the threshold were counted as pS6^+^ cells. SOX2^+^, Ki67^+^, PAX6^+^, CTIP2^+^ and BrdU^+^ cells were counted in the same way as pS6^+^ cells. All quantitative analyses were performed with at least three pairs of wild-type and CKO animals.

### *In utero* electroporation

*In utero* electroporation experiments followed guidelines from a previously published protocol (Baumgart and Baumgart, 2016). Timed pregnant C57BL/6 mice at gestation day 14.5 (E14.5) were anesthetized with isoflurane (induction in a chamber: 2.8%; via a mask during surgery: 2.5%). The uterine horns were exposed, and 0.5-1 µl of DNA plasmids (1-3 µg/µl) mixed with 5% Fast Green (Sigma-Aldrich) was slowly injected into the lateral ventricles of embryos using borosilicate capillaries (Drummond Scientific). Five 40-50 mV pulses (interval cycle length 50 ms, interval pause 950 ms) were then applied to the head of embryos via specialized forceps-type platinum electrodes (Nepa Gene, CUY650P5) to transfect the DG area. Animals were taken care of following standard post-electroporation and post-operative procedures (Baumgart and Baumgart, 2016) and closely monitored until giving birth. Brains of newborn pups were collected immediately for immunohistochemistry.

### Intracerebroventricular viral injection of neonatal mice

Intracerebroventricular viral injection of P0 *Ube4b^f/f^; Cas9/Cas9* littermates was performed as previously described (Kim et al., 2014). One newborn pup was transferred onto a cold plate to induce hypothermia anesthesia. Free-hand intracranial injection of 1 μl AAV virus mixture (∼10^9^ virus particles per hemisphere) was then performed after the pup became fully anesthetized. AAV viruses used in this study were: pAAV2/9-hSyn1-GFP, pAAV2/9-hSyn1-cre-P2A-GFP and pAAV2/2-CAG-KLHL22 sgRNAs. Two weeks post-injection, brains of injected pups were collected for biochemistry and immunohistochemistry.

### Label-free quantitative mass spectrometry

Whole brains from *Ube4b^f/f^* and *Ube4b Nestin*-CKO animals at P0 and P7 and forebrains from *Ube4b^f/f^* and *Ube4b Camk2a*-CKO animals at 7, 9 and 11 months were collected and frozen in liquid nitrogen immediately. Frozen brains were homogenized with mammalian cell lysis buffer (0.5% NP-40, 150 mM NaCl, 50 mM Tris, pH 7.8, 10 mM *N*-ethylmaleimide, supplied with protease and phosphatase inhibitors) at a ratio of 1:10 (w/v). After clarification at 17,000 ***g*** for 10 min at 4°C, equal amounts (200 µg) of *Ube4b^f/f^* and CKO brain lysate were in-gel digested with Trypsin overnight at 37°C and desalted using the C18 stage-tip method. Eluted peptides were analyzed using by liquid chromatography-mass spectrometry using a Q Exactive HF-X hybrid Quadrupole-Orbitrap (Thermo Scientific). A 1-h method with a 5-45% acetonitrile gradient was used.

Data from three pairs of littermates at each stage were used for label-free mass spectrometry analysis using MaxQuant (https://maxquant.org/maxquant/; version 1.6.2.0). The following parameters were used: carbamidomethyl of cysteine as fixed modification; main search peptide of 10 ppm; label-free quantification enabled. The reference database was downloaded from UniProtKB (http://www.uniprot.org/; accessed November 2019) for recommended protein names, gene names and protein functions. For quality control, all known contaminants (i.e. keratins, trypsin) and proteins detected in less than half of the samples were removed from each sample set of proteins identified. Up- and downregulated proteins were identified with adjusted label-free quantification (LFQ) intensities ratio with the following formula:

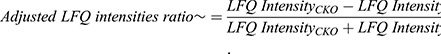
A protein with an adjusted LFQ intensities ratio ≥0.2 was considered upregulated. A protein with an adjusted LFQ intensities ratio ≤−0.15 was considered downregulated. Additionally, in any given age group in Fig. 2, only proteins with a peptide number greater than five and an increase in both absolute intensity and LFQ intensity were plotted to minimize false positives.

The DAVID analytic tool (https://david.ncifcrf.gov/) was used for Gene Ontology (GO) enrichment analysis. Fisher's Exact test was used for statistical analysis, corrected by Benjamini–Hochberg method. The threshold of false discovery rate (FDR) was set to 0.05. The GO knowledgebase of *Mus musculus* referenced in this experiment was released in December 2021 (DAVID 2021 with DAVID Knowledgebase v2021q4). Visualization of GO results was performed using R version 4.0.4. Raw scores of adjusted LFQ intensities ratios were normalized to Z-scores as below.

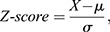
where: μ is the mean value of the population and σ is the standard deviation of the population.

The GO terms were plotted against counts, with a maximum of 15 GO terms displayed in each experimental group.

### Quantification and statistical analysis

For animal experiments, the numbers of animals are indicated in the text or present in the figures. Data represent mean± s.e.m. Comparisons between two groups were performed with two-tailed Student's *t*-test and comparisons among three or more groups were performed with one-way ANOVA using Graph Prism 9.

## Supplementary Material

10.1242/develop.201286_sup1 Supplementary information

## References

[DEV201286C1] Ackermann, L., Schell, M., Pokrzywa, W., Kevei, É., Gartner, A., Schumacher, B. and Hoppe, T. (2016). E4 ligase-specific ubiquitination hubs coordinate DNA double-strand-break repair and apoptosis. *Nat. Struct. Mol. Biol.* 23, 995-1002. 10.1038/nsmb.3296 27669035 PMC5349472

[DEV201286C2] Altman, J. and Bayer, S. A. (1990). Migration and distribution of two populations of hippocampal granule cell precursors during the perinatal and postnatal periods. *J. Comp. Neurol.* 301, 365-381. 10.1002/cne.903010304 2262596

[DEV201286C3] Antoniou, N., Lagopati, N., Balourdas, D. I., Nikolaou, M., Papalampros, A., Vasileiou, P. VS., Myrianthopoulos, V., Kotsinas, A., Shiloh, Y., Liontos, M. et al. (2019). The Role of E3, E4 Ubiquitin Ligase (UBE4B) in Human Pathologies. *Cancers (Basel)* 12, 62-76. 10.3390/cancers12010062 31878315 PMC7017255

[DEV201286C4] Bandeira, F., Lent, R. and Herculano-Houzel, S. (2009). Changing numbers of neuronal and non-neuronal cells underlie postnatal brain growth in the rat. *Proc. Natl. Acad. Sci. USA* 106, 14108-14113. 10.1073/pnas.0804650106 19666520 PMC2729028

[DEV201286C5] Bar-Peled, L., Chantranupong, L., Cherniack, A. D., Chen, W. W., Ottina, K. A., Grabiner, B. C., Spear, E. D., Carter, S. L., Meyerson, M. and Sabatini, D. M. (2013). A Tumor suppressor complex with GAP activity for the Rag GTPases that signal amino acid sufficiency to mTORC1. *Science* 340, 1100-1106. 10.1126/science.1232044 23723238 PMC3728654

[DEV201286C6] Baumgart, J. and Baumgart, N. (2016). Cortex-, hippocampus-, thalamus-, hypothalamus-, lateral septal nucleus- and striatum-specific in utero electroporation in the C57BL/6 mouse. *J. Vis. Exp.* 107, e53303. 10.3791/53303 PMC4781618 26862715

[DEV201286C7] Bond, A. M., Berg, D. A., Lee, S., Garcia-Epelboim, A. S., Adusumilli, V. S., Ming, G. L. and Song, H. (2020). Differential timing and coordination of neurogenesis and astrogenesis in developing mouse hippocampal subregions. *Brain Sci.* 10, 909. 10.3390/brainsci10120909 33255945 PMC7760658

[DEV201286C8] Carson, R. P., Van Nielen, D. L., Winzenburger, P. A. and Ess, K. C. (2012). Neuronal and glia abnormalities in Tsc1-deficient forebrain and partial rescue by rapamycin. *Neurobiol. Dis.* 45, 369-380. 10.1016/j.nbd.2011.08.024 21907282 PMC3225598

[DEV201286C9] Chen, J., Ou, Y., Yang, Y., Li, W., Xu, Y., Xie, Y. and Liu, Y. (2018). KLHL22 activates amino-acid-dependent mTORC1 signalling to promote tumorigenesis and ageing. *Nature* 557, 585-589. 10.1038/s41586-018-0128-9 29769719

[DEV201286C10] Cloëtta, D., Thomanetz, V., Baranek, C., Lustenberger, R. M., Lin, S., Oliveri, F., Atanasoski, S. and Rüegg, M. A. (2013). Inactivation of mTORC1 in the developing brain causes microcephaly and affects gliogenesis. *J. Neurosci.* 33, 7799-7810. 10.1523/JNEUROSCI.3294-12.2013 23637172 PMC6618947

[DEV201286C11] Crino, P. B. (2015). mTOR signaling in epilepsy: insights from malformations of cortical development. *Cold Spring Harb. Perspect. Med.* 5, a022442. 10.1101/cshperspect.a022442 25833943 PMC4382721

[DEV201286C12] Crowell, B., Lee, G. H., Nikolaeva, I., Dal Pozzo, V. and D'Arcangelo, G. (2015). Complex neurological phenotype in mutant mice lacking Tsc2 in excitatory neurons of the developing forebrain. *eNeuro* 2, e0046-e0060. 10.1523/ENEURO.0046-15.2015 PMC4676199 26693177

[DEV201286C13] Curatolo, P., Moavero, R. and de Vries, P. J. (2015). Neurological and neuropsychiatric aspects of tuberous sclerosis complex. *Lancet Neurol.* 14, 733-745. 10.1016/S1474-4422(15)00069-1 26067126

[DEV201286C14] De Fusco, A., Cerullo, M. S., Marte, A., Michetti, C., Romei, A., Castroflorio, E., Baulac, S. and Benfenati, F. (2020). Acute knockdown of Depdc5 leads to synaptic defects in mTOR-related epileptogenesis. *Neurobiol. Dis.* 139, 104822. 10.1016/j.nbd.2020.104822 32113911

[DEV201286C15] Dibbens, L. M., de Vries, B., Donatello, S., Heron, S. E., Hodgson, B. L., Chintawar, S., Crompton, D. E., Hughes, J. N., Bellows, S. T., Klein, K. M. et al. (2013). Mutations in DEPDC5 cause familial focal epilepsy with variable foci. *Nat. Genet.* 45, 546-551. 10.1038/ng.2599 23542697

[DEV201286C16] Ehninger, D., Han, S., Shilyansky, C., Zhou, Y., Li, W., Kwiatkowski, D. J., Ramesh, V. and Silva, A. J. (2008). Reversal of learning deficits in a Tsc2^+/−^ mouse model of tuberous sclerosis. *Nat. Med.* 14, 843-848. 10.1038/nm1788 18568033 PMC2664098

[DEV201286C17] Eriksson, P. S., Perfilieva, E., Björk-Eriksson, T., Alborn, A.-M., Nordborg, C., Peterson, D. A. and Gage, F. H. (1998). Neurogenesis in the adult human hippocampus. *Nat. Med.* 4, 1313-1317. 10.1038/3305 9809557

[DEV201286C18] Gabrielsen, M., Buetow, L., Nakasone, M. A., Ahmed, S. F., Sibbet, G. J., Smith, B. O., Zhang, W., Sidhu, S. S. and Huang, D. T. (2017). A general strategy for discovery of inhibitors and activators of RING and U-box E3 ligases with ubiquitin variants. *Mol. Cell* 68, 456-470.e410. 10.1016/j.molcel.2017.09.027 29053960 PMC5655547

[DEV201286C19] Gireud-Goss, M., Reyes, S., Tewari, R., Patrizz, A., Howe, M. D., Kofler, J., Waxham, M. N., McCullough, L. D. and Bean, A. J. (2020). The ubiquitin ligase UBE4B regulates amyloid precursor protein ubiquitination, endosomal trafficking, and amyloid β42 generation and secretion. *Mol. Cell. Neurosci.* 108, 103542. 10.1016/j.mcn.2020.103542 32841720 PMC7530133

[DEV201286C20] Gu, X., Orozco, J. M., Saxton, R. A., Condon, K. J., Liu, G. Y., Krawczyk, P. A., Scaria, S. M., Harper, J. W., Gygi, S. P. and Sabatini, D. M. (2017). SAMTOR is an S-adenosylmethionine sensor for the mTORC1 pathway. *Science* 358, 813-818. 10.1126/science.aao3265 29123071 PMC5747364

[DEV201286C21] Hana, S., Peterson, M., McLaughlin, H., Marshall, E., Fabian, A. J., McKissick, O., Koszka, K., Marsh, G., Craft, M., Xu, S. et al. (2021). Highly efficient neuronal gene knockout in vivo by CRISPR-Cas9 via neonatal intracerebroventricular injection of AAV in mice. *Gene Ther.* 28, 646-658. 10.1038/s41434-021-00224-2 33558692 PMC8599009

[DEV201286C22] Hatakeyama, S., Yada, M., Matsumoto, M., Ishida, N. and Nakayama, K.-I. (2001). U box proteins as a new family of ubiquitin-protein ligases. *J. Biol. Chem.* 276, 33111-33120. 10.1074/jbc.M102755200 11435423

[DEV201286C23] Hellerschmied, D., Roessler, M., Lehner, A., Gazda, L., Stejskal, K., Imre, R., Mechtler, K., Dammermann, A. and Clausen, T. (2018). UFD-2 is an adaptor-assisted E3 ligase targeting unfolded proteins. *Nat. Commun.* 9, 484. 10.1038/s41467-018-02924-7 29396393 PMC5797217

[DEV201286C24] Hoppe, T., Cassata, G., Barral, J. M., Springer, W., Hutagalung, A. H., Epstein, H. F. and Baumeister, R. (2004). Regulation of the myosin-directed chaperone UNC-45 by a novel E3/E4-multiubiquitylation complex in C. elegans. *Cell* 118, 337-349. 10.1016/j.cell.2004.07.014 15294159

[DEV201286C25] Hosoda, M., Ozaki, T., Miyazaki, K., Hayashi, S., Furuya, K., Watanabe, K.-I., Nakagawa, T., Hanamoto, T., Todo, S. and Nakagawara, A. (2005). UFD2a mediates the proteasomal turnover of p73 without promoting p73 ubiquitination. *Oncogene* 24, 7156-7169. 10.1038/sj.onc.1208872 16170377

[DEV201286C26] Inoki, K., Li, Y., Xu, T. and Guan, K.-L. (2003a). Rheb GTPase is a direct target of TSC2 GAP activity and regulates mTOR signaling. *Genes Dev.* 17, 1829-1834. 10.1101/gad.1110003 12869586 PMC196227

[DEV201286C27] Inoki, K., Zhu, T. and Guan, K.-L. (2003b). TSC2 mediates cellular energy response to control cell growth and survival. *Cell* 115, 577-590. 10.1016/S0092-8674(03)00929-2 14651849

[DEV201286C28] Janiesch, P. C., Kim, J., Mouysset, J., Barikbin, R., Lochmüller, H., Cassata, G., Krause, S. and Hoppe, T. (2007). The ubiquitin-selective chaperone CDC-48/p97 links myosin assembly to human myopathy. *Nat. Cell Biol.* 9, 379-390. 10.1038/ncb1554 17369820

[DEV201286C29] Jordan, V. K., Zaveri, H. P. and Scott, D. A. (2015). 1p36 deletion syndrome: an update. *Appl. Clin. Genet.* 8, 189-200. 10.2147/TACG.S65698 26345236 PMC4555966

[DEV201286C30] Kaneko, C., Hatakeyama, S., Matsumoto, M., Yada, M., Nakayama, K. and Nakayama, K. I. (2003). Characterization of the mouse gene for the U-box-type ubiquitin ligase UFD2a. *Biochem. Biophys. Res. Commun.* 300, 297-304. 10.1016/S0006-291X(02)02834-6 12504083

[DEV201286C31] Kaneko-Oshikawa, C., Nakagawa, T., Yamada, M., Yoshikawa, H., Matsumoto, M., Yada, M., Hatakeyama, S., Nakayama, K. and Nakayama, K. I. (2005). Mammalian E4 is required for cardiac development and maintenance of the nervous system. *Mol. Cell. Biol.* 25, 10953-10964. 10.1128/MCB.25.24.10953-10964.2005 16314518 PMC1316961

[DEV201286C32] Kim, E., Goraksha-Hicks, P., Li, L., Neufeld, T. P. and Guan, K.-L. (2008). Regulation of TORC1 by Rag GTPases in nutrient response. *Nat. Cell Biol.* 10, 935-945. 10.1038/ncb1753 18604198 PMC2711503

[DEV201286C33] Kim, J.-Y., Grunke, S. D., Levites, Y., Golde, T. E. and Jankowsky, J. L. (2014). Intracerebroventricular viral injection of the neonatal mouse brain for persistent and widespread neuronal transduction. *J. Vis. Exp.* 91, 51863. 10.3791/51863 PMC4199253 25286085

[DEV201286C34] Koegl, M., Hoppe, T., Schlenker, S., Ulrich, H. D., Mayer, T. U. and Jentsch, S. (1999). A novel ubiquitination factor, E4, is involved in multiubiquitin chain assembly. *Cell* 96, 635-644. 10.1016/S0092-8674(00)80574-7 10089879

[DEV201286C35] Li, G. and Pleasure, S. J. (2005). Morphogenesis of the dentate gyrus: what we are learning from mouse mutants. *Dev. Neurosci.* 27, 93-99. 10.1159/000085980 16046842

[DEV201286C36] Liu, G. Y. and Sabatini, D. M. (2020). mTOR at the nexus of nutrition, growth, ageing and disease. *Nat. Rev. Mol. Cell Biol.* 21, 183-203. 10.1038/s41580-019-0199-y 31937935 PMC7102936

[DEV201286C37] Magri, L., Cambiaghi, M., Cominelli, M., Alfaro-Cervello, C., Cursi, M., Pala, M., Bulfone, A., Garcìa-Verdugo, J. M., Leocani, L., Minicucci, F. et al. (2011). Sustained activation of mTOR pathway in embryonic neural stem cells leads to development of tuberous sclerosis complex-associated lesions. *Cell Stem Cell* 9, 447-462. 10.1016/j.stem.2011.09.008 22056141

[DEV201286C38] Matsumoto, M., Yada, M., Hatakeyama, S., Ishimoto, H., Tanimura, T., Tsuji, S., Kakizuka, A., Kitagawa, M. and Nakayama, K. I. (2004). Molecular clearance of ataxin-3 is regulated by a mammalian E4. *EMBO J.* 23, 659-669. 10.1038/sj.emboj.7600081 14749733 PMC1271811

[DEV201286C39] Meikle, L., Talos, D. M., Onda, H., Pollizzi, K., Rotenberg, A., Sahin, M., Jensen, F. E. and Kwiatkowski, D. J. (2007). A mouse model of tuberous sclerosis: neuronal loss of Tsc1 causes dysplastic and ectopic neurons, reduced myelination, seizure activity, and limited survival. *J. Neurosci.* 27, 5546-5558. 10.1523/JNEUROSCI.5540-06.2007 17522300 PMC6672762

[DEV201286C40] Memarzadeh, K., Savage, D. J. and Bean, A. J. (2019). Low UBE4B expression increases sensitivity of chemoresistant neuroblastoma cells to EGFR and STAT5 inhibition. *Cancer Biol. Ther.* 20, 1416-1429. 10.1080/15384047.2019.1647049 31475882 PMC6804809

[DEV201286C41] Menon, S., Dibble, C. C., Talbott, G., Hoxhaj, G., Valvezan, A. J., Takahashi, H., Cantley, L. C. and Manning, B. D. (2014). Spatial control of the TSC complex integrates insulin and nutrient regulation of mTORC1 at the lysosome. *Cell* 156, 771-785. 10.1016/j.cell.2013.11.049 24529379 PMC4030681

[DEV201286C42] Ohi, M. D., Vander Kooi, C. W., Rosenberg, J. A., Chazin, W. J. and Gould, K. L. (2003). Structural insights into the U-box, a domain associated with multi-ubiquitination. *Nat. Struct. Biol.* 10, 250-255. 10.1038/nsb906 12627222 PMC5881891

[DEV201286C43] Parenti, I., Rabaneda, L. G., Schoen, H. and Novarino, G. (2020). Neurodevelopmental Disorders: From Genetics to Functional Pathways. *Trends Neurosci.* 43, 608-621. 10.1016/j.tins.2020.05.004 32507511

[DEV201286C44] Paridaen, J. T. and Huttner, W. B. (2014). Neurogenesis during development of the vertebrate central nervous system. *EMBO Rep.* 15, 351-364. 10.1002/embr.201438447 24639559 PMC3989667

[DEV201286C45] Ribierre, T., Deleuze, C., Bacq, A., Baldassari, S., Marsan, E., Chipaux, M., Muraca, G., Roussel, D., Navarro, V., Leguern, E. et al. (2018). Second-hit mosaic mutation in mTORC1 repressor DEPDC5 causes focal cortical dysplasia-associated epilepsy. *J. Clin. Invest.* 128, 2452-2458. 10.1172/JCI99384 29708508 PMC5983335

[DEV201286C46] Scharfman, H. E. (2019). The dentate gyrus and temporal lobe epilepsy: an “exciting” era. *Epilepsy Curr.* 19, 249-255. 10.1177/1535759719855952 31232111 PMC6891841

[DEV201286C47] Sirisaengtaksin, N., Gireud, M., Yan, Q., Kubota, Y., Meza, D., Waymire, J. C., Zage, P. E. and Bean, A. J. (2014). UBE4B protein couples ubiquitination and sorting machineries to enable epidermal growth factor receptor (EGFR) degradation. *J. Biol. Chem.* 289, 3026-3039. 10.1074/jbc.M113.495671 24344129 PMC3908433

[DEV201286C48] Starita, L. M., Pruneda, J. N., Lo, R. S., Fowler, D. M., Kim, H. J., Hiatt, J. B., Shendure, J., Brzovic, P. S., Fields, S. and Klevit, R. E. (2013). Activity-enhancing mutations in an E3 ubiquitin ligase identified by high-throughput mutagenesis. *Proc. Natl. Acad. Sci. USA* 110, E1263-E1272. 10.1073/pnas.1303309110 23509263 PMC3619334

[DEV201286C49] Sugiyama, T., Osumi, N. and Katsuyama, Y. (2013). The germinal matrices in the developing dentate gyrus are composed of neuronal progenitors at distinct differentiation stages. *Dev. Dyn.* 242, 1442-1453. 10.1002/dvdy.24035 24038449

[DEV201286C50] Tronche, F., Kellendonk, C., Kretz, O., Gass, P., Anlag, K., Orban, P. C., Bock, R., Klein, R. and Schütz, G. (1999). Disruption of the glucocorticoid receptor gene in the nervous system results in reduced anxiety. *Nat. Genet.* 23, 99-103. 10.1038/12703 10471508

[DEV201286C51] Tsien, J. Z., Chen, D. F., Gerber, D., Tom, C., Mercer, E. H., Anderson, D. J., Mayford, M., Kandel, E. R. and Tonegawa, S. (1996). Subregion- and cell type-restricted gene knockout in mouse brain. *Cell* 87, 1317-1326. 10.1016/S0092-8674(00)81826-7 8980237

[DEV201286C52] Valvezan, A. J. and Manning, B. D. (2019). Molecular logic of mTORC1 signalling as a metabolic rheostat. *Nat. Metab.* 1, 321-333. 10.1038/s42255-019-0038-7 32694720 PMC12569966

[DEV201286C53] Wang, Z., Edwards, J. G., Riley, N., Provance, D. W., Jr, Karcher, R., Li, X. D., Davison, I. G., Ikebe, M., Mercer, J. A., Kauer, J. A. et al. (2008). Myosin Vb mobilizes recycling endosomes and AMPA receptors for postsynaptic plasticity. *Cell* 135, 535-548. 10.1016/j.cell.2008.09.057 18984164 PMC2585749

[DEV201286C54] Wang, Z., Hou, Y., Guo, X., van der Voet, M., Boxem, M., Dixon, J. E., Chisholm, A. D. and Jin, Y. (2013). The EBAX-type Cullin-RING E3 ligase and Hsp90 guard the protein quality of the SAX-3/Robo receptor in developing neurons. *Neuron* 79, 903-916. 10.1016/j.neuron.2013.06.035 24012004 PMC3779136

[DEV201286C55] Wang, G., Lei, J., Wang, Y., Yu, J., He, Y., Zhao, W., Hu, Z., Xu, Z., Jin, Y., Gu, Y. et al. (2022). The ZSWIM8 ubiquitin ligase regulates neurodevelopment by guarding the protein quality of intrinsically disordered Dab1. *Cereb. Cortex*, bhac313. 10.1093/cercor/bhac313 35989311

[DEV201286C56] Wu, H., Pomeroy, S. L., Ferreira, M., Teider, N., Mariani, J., Nakayama, K. I., Hatakeyama, S., Tron, V. A., Saltibus, L. F., Spyracopoulos, L. et al. (2011). UBE4B promotes Hdm2-mediated degradation of the tumor suppressor p53. *Nat. Med.* 17, 347-355. 10.1038/nm.2283 21317885

[DEV201286C57] Xiong, Y., Zhang, Y., Xiong, S. and Williams-Villalobo, A. E. (2020). A glance of p53 functions in brain development, neural stem cells, and brain cancer. *Biology (Basel)* 9, 285. 10.3390/biology9090285 32932978 PMC7564678

[DEV201286C58] Yuskaitis, C. J., Jones, B. M., Wolfson, R. L., Super, C. E., Dhamne, S. C., Rotenberg, A., Sabatini, D. M., Sahin, M. and Poduri, A. (2018). A mouse model of DEPDC5-related epilepsy: neuronal loss of Depdc5 causes dysplastic and ectopic neurons, increased mTOR signaling, and seizure susceptibility. *Neurobiol. Dis.* 111, 91-101. 10.1016/j.nbd.2017.12.010 29274432 PMC5803417

[DEV201286C59] Zage, P. E., Sirisaengtaksin, N., Liu, Y., Gireud, M., Brown, B. S., Palla, S., Richards, K. N., Hughes, D. P. and Bean, A. J. (2013). UBE4B levels are correlated with clinical outcomes in neuroblastoma patients and with altered neuroblastoma cell proliferation and sensitivity to epidermal growth factor receptor inhibitors. *Cancer* 119, 915-923. 10.1002/cncr.27785 22990745 PMC3527637

[DEV201286C60] Zhou, J., Shrikhande, G., Xu, J., McKay, R. M., Burns, D. K., Johnson, J. E. and Parada, L. F. (2011). Tsc1 mutant neural stem/progenitor cells exhibit migration deficits and give rise to subependymal lesions in the lateral ventricle. *Genes Dev.* 25, 1595-1600. 10.1101/gad.16750211 21828270 PMC3182017

