# Supplementary material for: Fine-tuning of mTOR signaling by the UBE4B-KLHL22 E3 ubiquitin ligase cascade in brain development
Source: Development. 2022 Dec 20;149(24):dev201286. doi: 10.1242/dev.201286 (PMC9845739; doi:10.1242/dev.201286)
Supplement: Supplementary information [file develop-149-201286-s1.pdf]

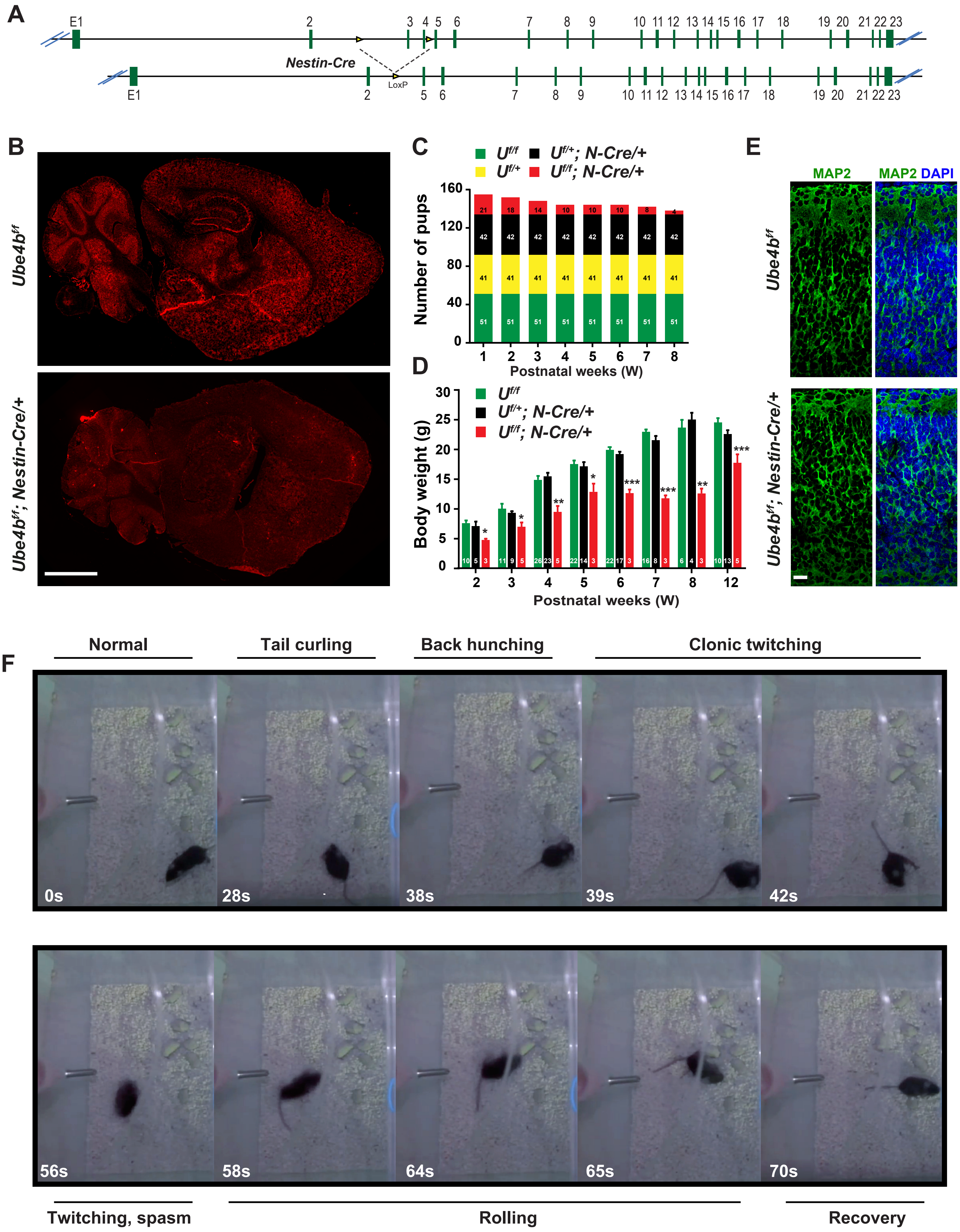

Development • Supplementary information

Fig. S1. *Ube4b Nestin*-CKO mice show developmental defects and epilepsy

(A) Schematic of the strategy for generating the *Ube4b floxed* allele and *Ube4b*$^{f/+}$*; Nestin-Cre/*+ mice. Two LoxP sites were inserted in introns 2 and 4 by CRISPR. *Ube4b*$^{f/f}$ and *Ube4b*$^{f/+}$*; Nestin-Cre/*+ were crossed to obtain heterozygous and homozygous conditional knockout progeny.

(B) Validation of *Ube4b* deletion by RNAscope in sagittal brain sections from a pair of *Ube4b*$^{f/f}$ (top) and *Ube4b Nestin*-CKO (bottom) littermates. Scale bar, 500 µm.

(C) Numbers of survivors of four genotypes bred by *Ube4b*$^{f/f}$ and *Ube4b*$^{f/+}$*; Nestin-Cre/*+ parents. Pups from a total of 16 litters were tracked from week 1 to week 8. N numbers for each genotype are marked on the graph. No death was observed for the first three genotypes in 8 weeks. In contrast, very few CKO animals survived to adulthood.

(D) Body weight of progeny born by *Ube4b*$^{f/f}$ and *Ube4b*$^{f/+}$*; Nestin-Cre/*+ parents. N numbers for each genotype are marked on the graph. Data represent means ± SEM. *p < 0.05, **p < 0.01, and ***p < 0.001 relative to *Ube4b*$^{f/f}$ in the same age group; one-way ANOVA.

(E) Dendritic arborization shown by microtubule-associated protein 2 (MAP2) staining in P0 cortical slices. Disorganization of dendritic trees was noticed in the S1 cortex of CKO animals. Scale bar, 50 µm.

(F) Representative behaviors of a 1-month-old CKO mouse that was experiencing a tonic-clonic seizure. Onset of the seizure episode started with sudden body stiffness with curling tail and hunched back. Then the animal showed rhythmic twitching of its body and muscle contraction, followed by body rolling. At the end of the seizure, the animal became quiet for a few seconds and then crawled away (Recovery).

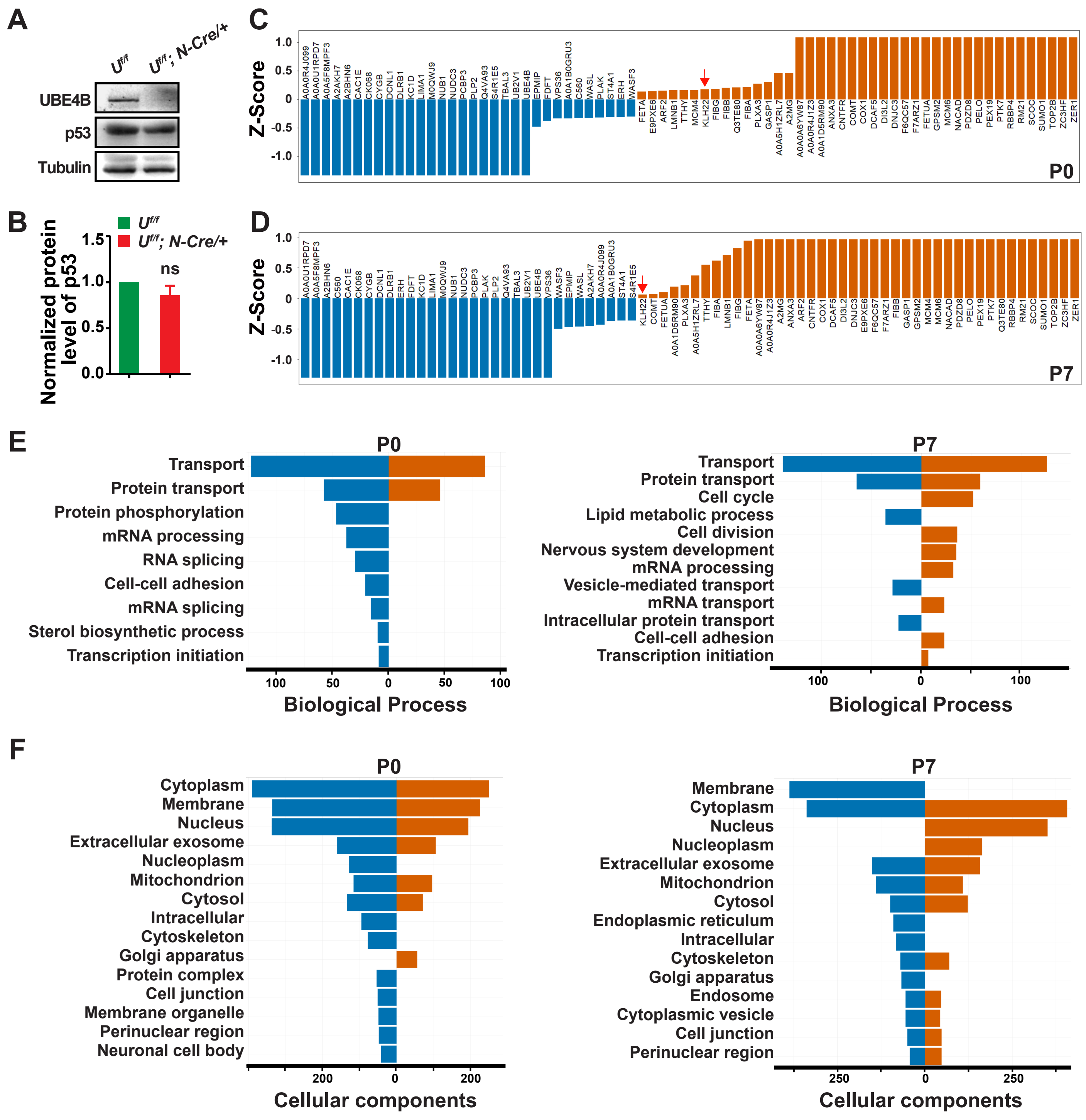

Fig. S2. Deletion of UBE4B in NSCs causes upregulation of KLHL22 but not p53

(A-B) The expression level of p53 was not significantly changed by UBE4B deletion in the brain. Representative blots from three pairs of *Ube4b*$^{f/f}$ and *Ube4B Nestin*-CKO littermates are shown in (A). Quantification analysis of p53 expression is shown in (B).

(C-D) Z-score histograms of upregulated and downregulated proteins in *Ube4B Nestin*-CKO brains at P0 (C) and P7 (D) stages. Adjusted relative LFQ intensity ratios of individual proteins from mass spectrometry were normalized into Z-score for alignment. Orange bars represent commonly upregulated proteins and blue bars represent commonly downregulated proteins. Red arrows indicate KLHL22. Selection criteria are described in the Method section.

(E-F) GO enrichment analysis of biological processes (E) and cellular components (F) on upregulated and downregulated proteins in P0 and P7 *Nestin*-CKO mice. Commonly upregulated and downregulated proteins are represented by orange and blue horizontal bars, respectively. The x-axis represents the protein count. Details are described in the Method session.

Development • Supplementary information

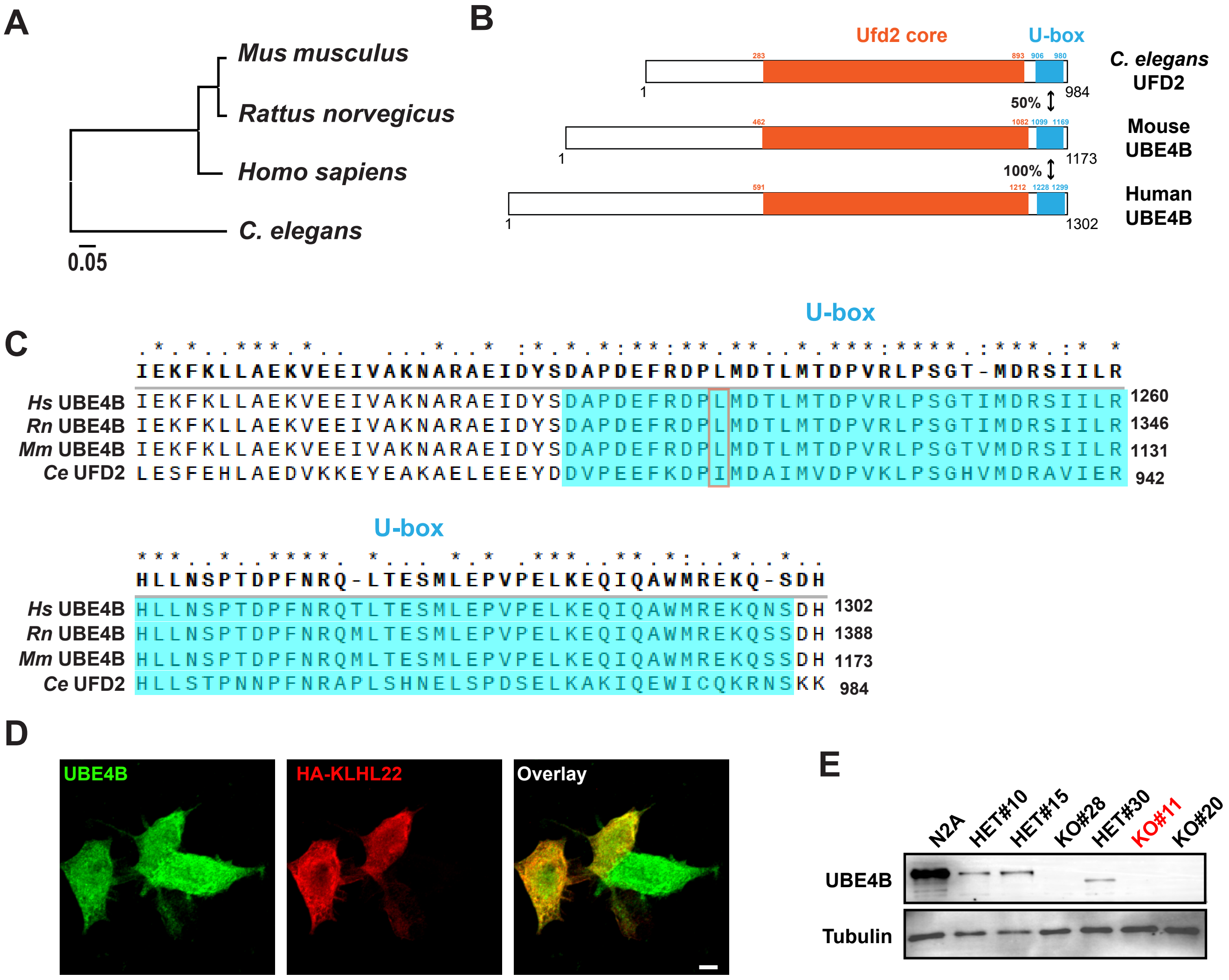

Fig. S3. Knockout of the U-box E3 ligase UBE4B in Neuro2A suppresses proliferation

(A) Phylogenetic tree of UBE4B homologs from different species.

(B) Schematic alignment of worm UFD2, mouse UBE4B and human UBE4B proteins. The starts and ends of Ufd2 core (orange) and U-box (blue) domains are marked by numbers. The percentages of sequence identity within these two domains between species are indicated.

(C) Sequence alignment of the U-box domains (highlighted in cyan) from different species. The leucine residue in the red box is conserved in human, mouse and rat but not*C. elegans*.

(D) Subcellular localization of endogenous UBE4B and exogenous HA-KLHL22 in Neuro2A cells. KLHL22 was detected by anti-HA antibodies. Scale bar, 10 μm. (E) A pair of sgRNAs targeting exons 4 and 5 of UBE4B were introduced into Neuro2A cells. Following drug selection, single clones were expanded and immunoblotted for UBE4B. γ-tubulin was used as the loading control. Multiple heterozygous and homozygous knockout clones were obtained. The KO#11 clone was used for the CHX experiment in Figure 3E.

Development • Supplementary information

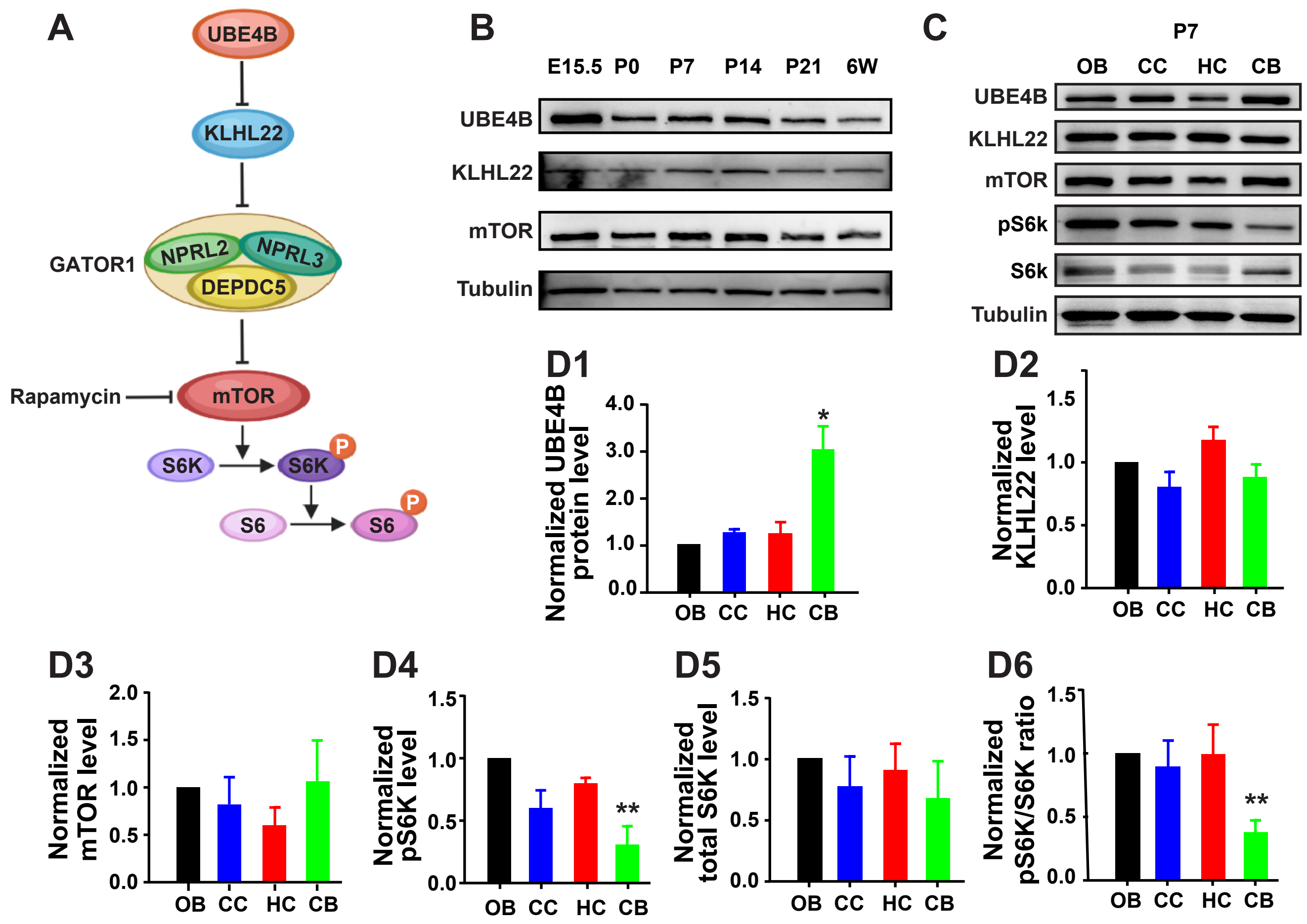

Fig. S4. Spatial and temporal expression of UBE4B, KLHL22 and components of the mTOR pathway in rodent brains

(A) Schematic model illustrating the regulation of mTOR signaling by the UBE4B-KLHL22 E3 cascade.

(B) Temporal expression of UBE4B, KLHL22 and mTOR in mouse brains starting from E15.5 to 6 weeks. γ-tubulin was used as the loading control.

(C) Spatial expression of UBE4B, KLHL22, mTOR, S6K and phosphorylated S6K in wildtype rat brains at P7. OB, olfactory bulb; CC, cerebral cortex; HC, hippocampus; CB, cerebellum.

(D1-D6) Quantification of UBE4B, KLHL22, mTOR, pS6K and S6K expression levels and normalized pS6K / S6K ratio in OB, CC, HC and CB from P7 rat brains (N = 3 biological replicates). Data represent means ± SEM. *$p < 0.05$ and **$p < 0.01$ relative to normalized protein level in OB; Student's *t*-test.

Development • Supplementary information

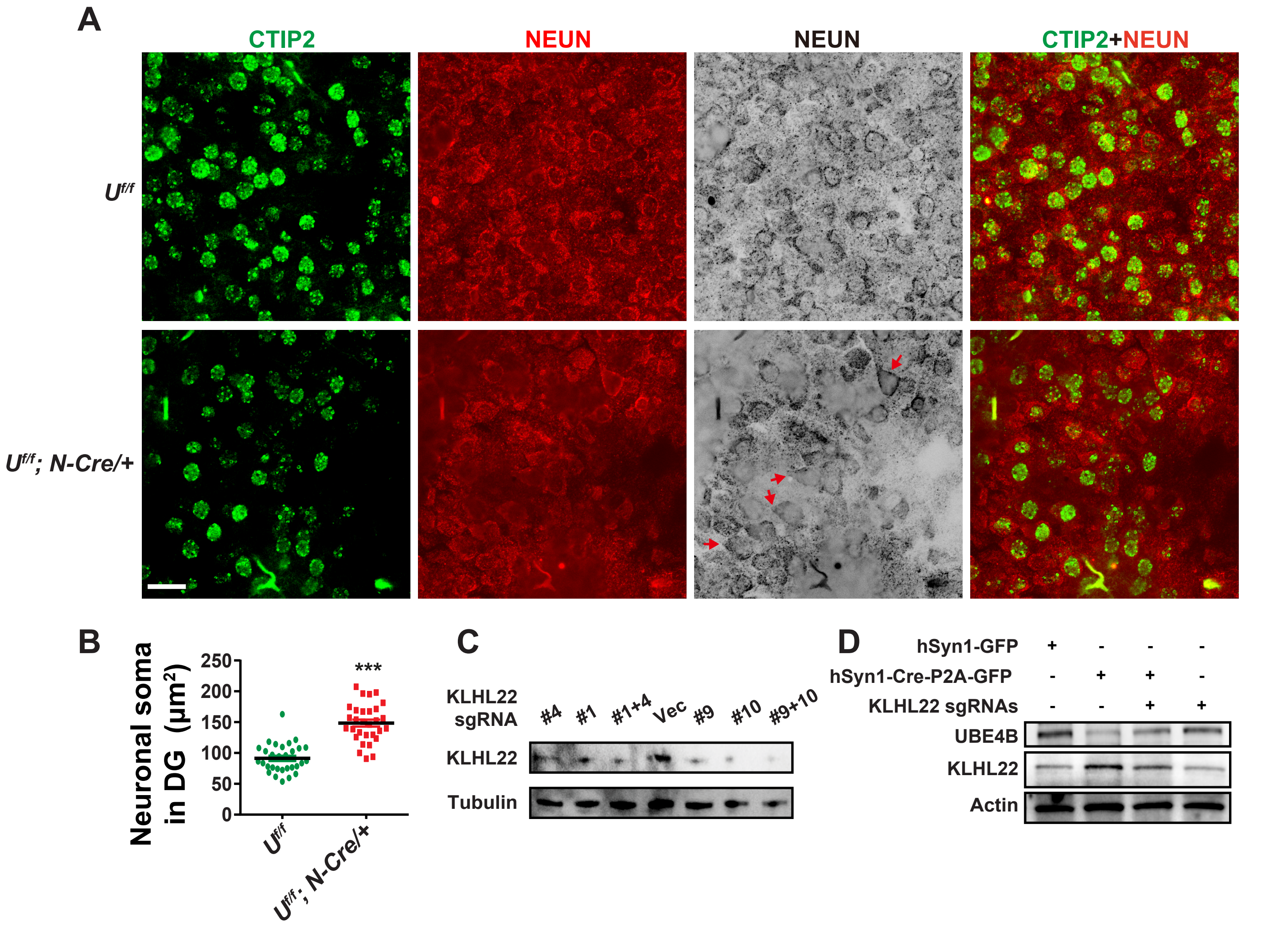

Fig. S5. The soma size of neurons in the forebrain is not affected by UBE4b deletion

(A-B) Immunohistochemistry of NeuN-labeled neurons in the DG of *Ube4b*$^{f/f}$ and CKO littermates at the P0 stage (A). CTIP2 was co-stained to label granular cells in the same regions. Arrows indicate neurons with enlarged soma. Scale bar, 20 μm. Quantification of the soma size of NeuN^{+} neurons in DG of *Ube4b*$^{f/f}$ and CKO littermates is shown in (B).

(C) CRISPR knockout efficiency examined by immunoblotting of endogenous KLHL22 in Neuro2A cells transfected with PX459 vector control (Vec), individual sgRNAs and sgRNA pairs targeting KLHL22. Transfected cells were selected by puromycin for three days before being collected for analysis. γ-tubulin was used as the loading control. The sgRNAs #1 and #4 were used for *in utero* electroporation experiments. The sgRNAs #9 and #10 were used for AAV viral expression experiments.

(D) Upregulation of KLHL22 by acute deletion of UBE4B in the postnatal brain is reversed by co-delivery of KLHL22 sgRNAs. AAV viruses carrying *hSyn-1-Cre-P2A-GFP* and KLHL22 sgRNAs were injected alone or together into the lateral ventricle of P0 *Ube4b*$^{f/f}$*; Cas9/Cas9* animals. pAAV2/9-hSyn1-GFP viruses was used as a negative control. Two weeks after injection, infected cortical tissues were collected and immunoblotted for UBE4B, KLHL22 and β-actin.

Development • Supplementary information

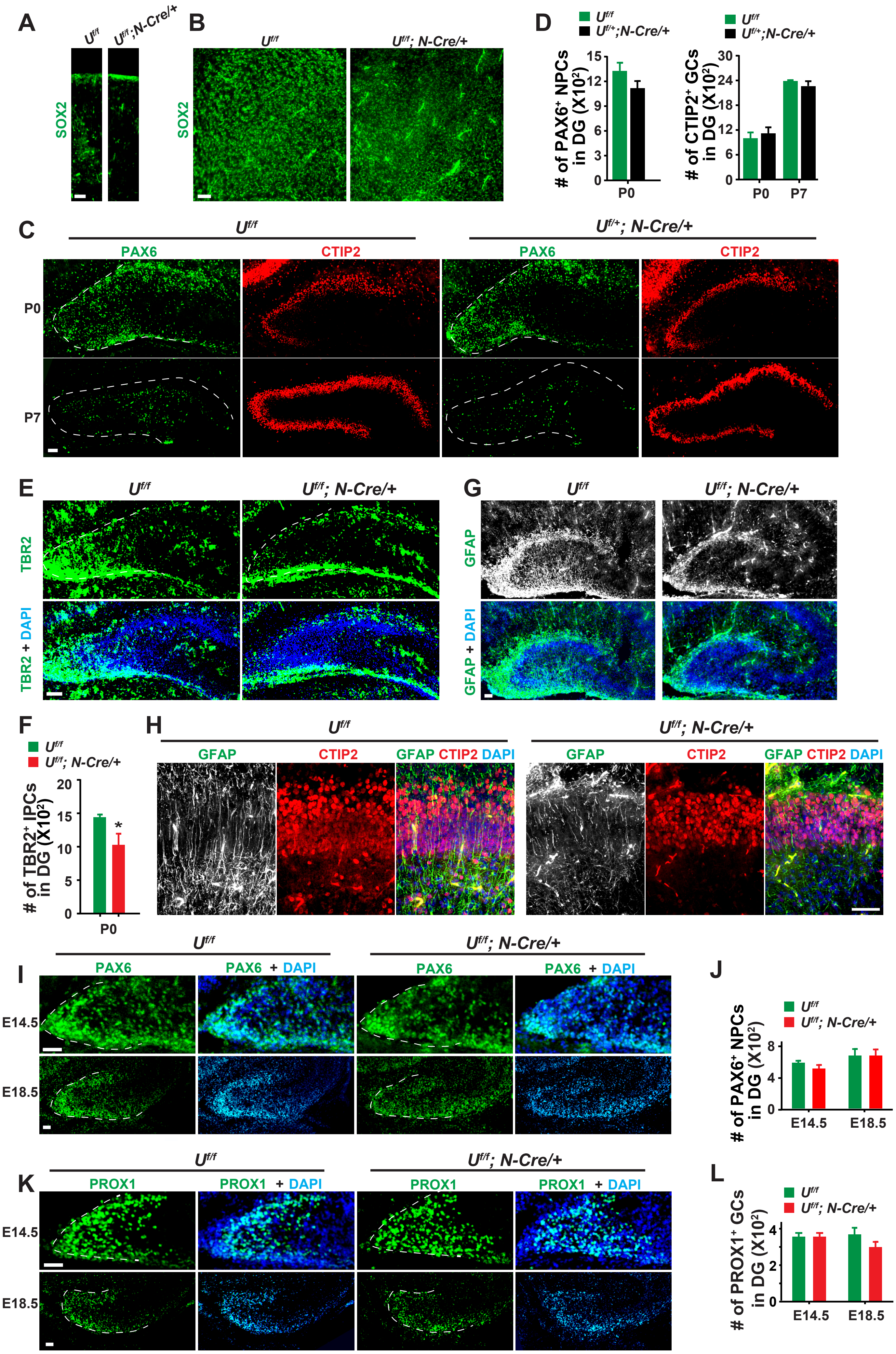

A
B
C
D
E
F
G
H
I
J
K
L
SOX2
PAX6
CTIP2
TBR2
TBR2 + DAPI
GFAP
GFAP + DAPI
GFAP CTIP2 DAPI
PAX6 + DAPI
PROX1
PROX1 + DAPI
P0
P7
E14.5
E18.5
# of PAX6⁺ NPCs in DG (X10²)
# of CTIP2⁺ GCs in DG (X10²)
# of TBR2⁺ IPCs in DG (X10²)
# of PROX1⁺ GCs in DG (X10²)

Fig. S6. The numbers of NPCs and GCs are not changed in embryonic *Ube4b Nestin*-CKO brains and postnatal heterozygous CKOs

(A-B) SOX2 immunohistochemistry in the S1 cerebral cortex (A) and in the midbrain (B). A noticeable reduction of SOX2 signals was observed in these regions. Scale bars, 50 μm.

(C-D) Immunohistochemistry and quantification of PAX6^{+} NPCs and CTIP2^{+} GCs in the DG of *Ube4b*$^{f/f}$ and heterozygous *Nestin*-CKO animals at P0 and P7 (C). Scale bars, 50 μm. Brain slices from three pairs of littermates at each stage were quantified in (D). Data represent means ± SEM. No significant difference was detected; Student's *t-test*.

(E-F) Immunohistochemistry of TBR2^{+} intermediate progenitors (IPs) in the DG from P0 *Ube4b*$^{f/f}$ and CKO animals (E). Scale bar, 50 μm. Brain slices from three pairs of P0 *Ube4b*$^{f/f}$ and CKO littermates were quantified in (F). Data represent means ± SEM. *p < 0.05 relative to *Ube4b*$^{f/f}$; Student's *t*-test.

(G-H) GFAP-labeled radial glial scaffold was disorganized in the hippocampus of CKO animals. Three pairs of P0 *Ube4b*$^{f/f}$ and CKO littermates were examined. Representative images of GFAP immunostaining in the whole DGs (G) and zoomed radial glial cells in (H) are demonstrated. Scale bars, 100 μm (G) and 50 μm (H).

(I-L) Representative images of PAX6^{+} NPCs (I) and PROX1^{+} GCs (K) in the DG of *Ube4b*$^{f/f}$ and homozygous *Nestin*-CKO animals at E14.5 and E18.5. Scale bars, 50 μm. Data in (J) and (L) represent means ± SEM from three pairs of littermates; Student's *t-test*.

Development • Supplementary information

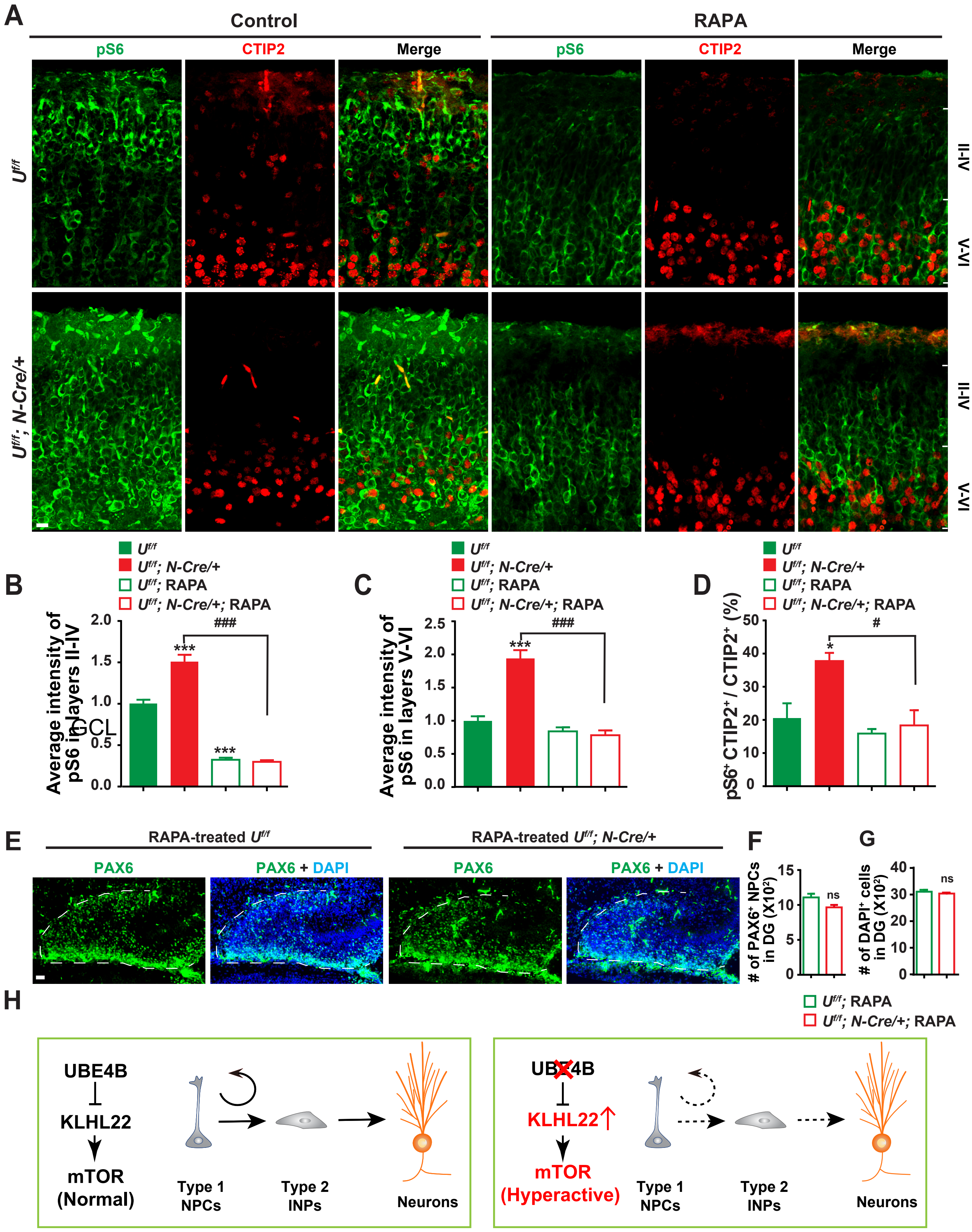

Development • Supplementary information

Fig. S7. Hyperactivation of mTOR in the cerebral cortex is reversed by prenatal treatment of rapamycin in UBE4B CKO animals

(A) Immunohistochemistry of phospho-S6 and CTIP2 in the S1 cerebral cortex of newborn *Ube4b*$^{f/f}$ and *Ube4b*$^{f/f}$*; Nestin-Cre/+* animals following the treatment

(control or rapamycin) paradigm in Figure 7A. The boundary between layers II-IV and layers V-VI was grossly defined by the density of CTIP2-marked cells. Scale bar, 50 μm.

(B-C) Quantification of average intensity of pS6 signals in the layers II-IV (B) and V-VI (C). Brain slides from 3 pairs of animals were quantified for the control and rapamycin groups, respectively. ***$p < 0.001$ relative to *Ube4b*$^{f/f}$ treated with vehicle control; $^{###}p < 0.001$ relative to CKO treated with vehicle control; one-way ANOVA.

(D) Effects of rapamycin treatment on the percentage of pS6^{+} CTIP2^{+} GCs in the S1 cortex. N = 3 animals per group. *$p < 0.05$ relative to *Ube4b*$^{f/f}$ treated with vehicle control; $^{\#}p < 0.05$ relative to CKO treated with vehicle control; one-way ANOVA.

(E-G) Immunohistochemistry of PAX6 in the DG of rapamycin-treated newborn *Ube4b*$^{f/f}$ and *Ube4b*$^{f/f}$*; Nestin-Cre/+* animals. Scale bar, 100 μm. No significant difference (ns) in the total numbers of PAX6^{+} NPCs (F) or DAPI-marked nuclei

(G) was observed between quantified littermates.

(H) A model of mTOR regulation by the UBE4B-KLHL22 E3 cascade. UBE4B restricts the level of KLHL22 and keeps the activity of mTOR at a level optimal for NPC proliferation and differentiation. In the absence of UBE4B, accumulated KLHL22 causes mTOR hyperactivation, which leads to impaired NPC proliferation and differentiation.

Table S1. List of antibodies, plasmids, mouse strains and software

Click here to download Table S1

Table S2. Lists of upregulated and downregulated proteins in P0, P7, 7 M, 9 M and 11 M brains.

Click here to download Table S2

Development • Supplementary information

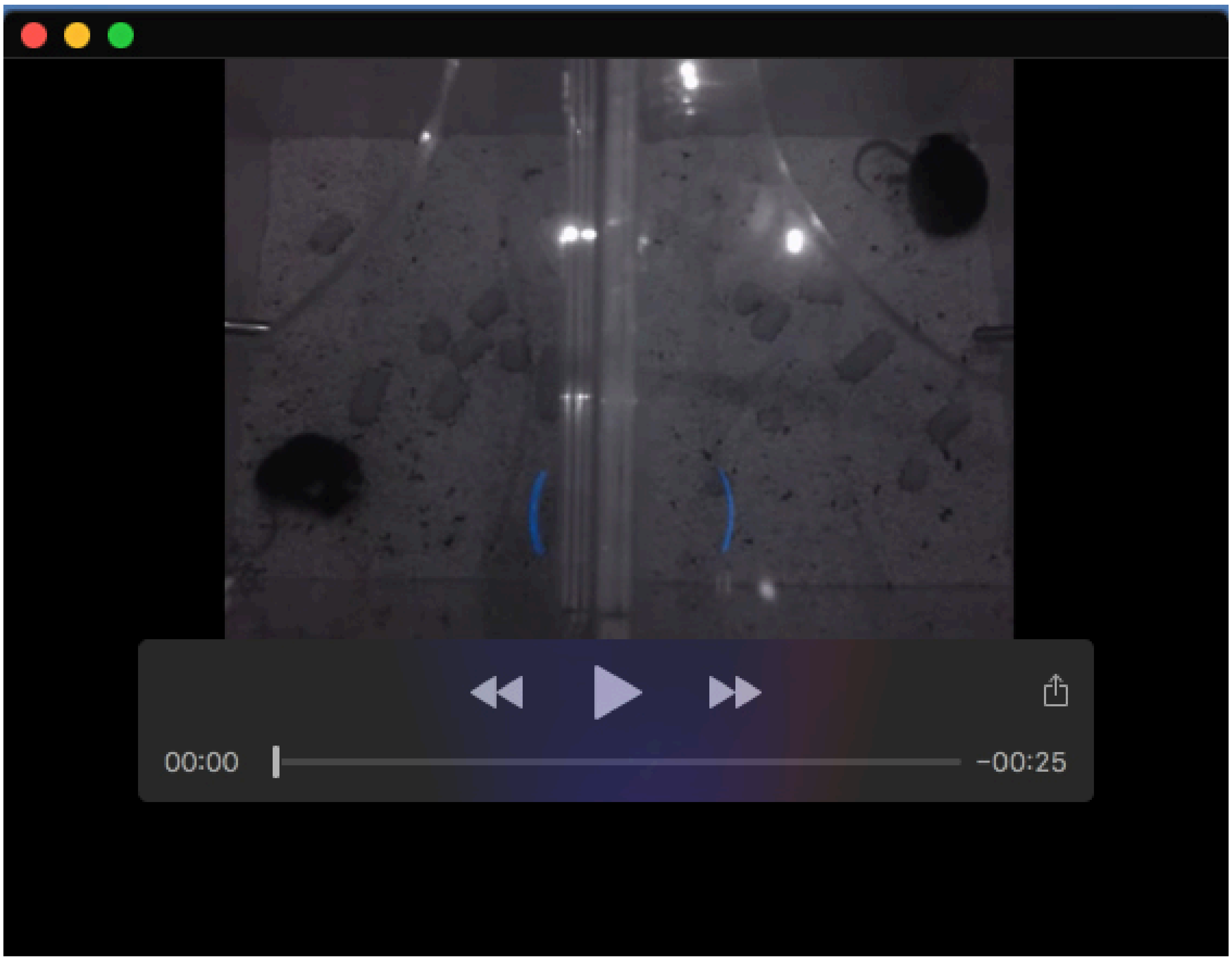

Movie 1. The video demonstrates an epileptic *Ube4b* CKO mouse (on the left) and its wild-type littermate (on the right) which were continuously recorded for their behaviors in a recording chamber. The seizure episode starts around 1 minute in the movie.

Development • Supplementary information
