# Supplementary material for: Fine-tuning of mTOR signaling by the UBE4B-KLHL22 E3 ubiquitin ligase cascade in brain development
Source: Development. 2022 Dec 20;149(24):dev201286. doi: 10.1242/dev.201286 (PMC9845739; doi:10.1242/dev.201286)
Supplement: Supplementary file 1 [file develop-149-201286_review_history.pdf]

Fine-tuning of mTOR signaling by the UBE4B-KLHL22 E3 ubiquitin ligase cascade in brain development

Xiangxing Kong, Xin Shu, Jiachuan Wang, Dandan Liu, Yingchun Ni, Weiqi Zhao, Lebo Wang, Zhihua Gao, Jiadong Chen, Bing Yang, Xing Guo and Zhiping Wang
DOI: 10.1242/dev.201286

Editor: Francois Guillemot

Review timeline
Original submission: 26 September 2022
Editorial decision: 20 October 2022
First revision received: 9 November 2022
Accepted: 15 November 2022

Original submission

First decision letter

MS ID#: DEVELOP/2022/201286

MS TITLE: Fine-tuning of mTOR signaling by the UBE4B-KLHL22 E3 ubiquitin ligase cascade in brain development

AUTHORS: Zhiping Wang, Xiangxing Kong, Xin Shu, Jiachuan Wang, Dandan Liu, Yingchun Ni, Weiqi Zhao, Zhihua Gao, Jiadong Chen, Xing Guo, and Bing Yang

I have now received the reports of two referees on your manuscript, and have reached a decision. The referees' comments are appended below, or you can access them online: please go to BenchPress and click on the 'Manuscripts with Decisions' queue in the Author Area.

As you will see, the referees express great interest in your work, but they also have significant criticisms and recommend a substantial revision of your manuscript before we can consider publication. In particular, referee 2 requests that you extend the analysis of UBE4 to earlier developmental stages . If you are able to revise the manuscript along the lines suggested by the referees, which may involve further experiments, I will be happy to receive a revised version of the manuscript. Your revised paper will be re-reviewed by one or more of the original referees, and acceptance of your manuscript will depend on your addressing satisfactorily the reviewers' major concerns. Please also note that Development will normally permit only one round of major revision. If it would be helpful, you are welcome to contact us to discuss your revision in greater detail. Please send us a point-by-point response indicating your plans for addressing the referee's comments, and we will look over this and provide further guidance.

Please attend to all of the reviewers' comments and ensure that you clearly highlight all changes made in the revised manuscript. Please avoid using 'Tracked changes' in Word files as these are lost in PDF conversion. I should be grateful if you would also provide a point-by-point response detailing how you have dealt with the points raised by the reviewers in the 'Response to Reviewers' box. If you do not agree with any of their criticisms or suggestions please explain clearly why this is so.

© 2022. Published by The Company of Biologists under the terms of the Creative Commons Attribution License (https://creativecommons.org/licenses/by/4.0/).

Reviewer 1

Advance summary and potential significance to field

This manuscript presents a careful and thorough examination of the role of a ubiquitin ligase system in brain development, mediated through the mTOR pathway. Elements of the pathway have been described before, but this manuscript presents a complete picture, systematically following through from biochemical studies all the way to a drug treatment that rescues the effect of their mutation. In my opinion, this represents a significant advance.

Comments for the author

The manuscript is clear and easy to follow, the data presented are of high quality and are convincing. I have only a few minor comments and suggestions for the authors.

Fig 1 legend says that hippocampus in panel 1G is highlighted by red rectangles, these are missing on my copy.

Analysis starts at E15.5, but many nestin expressing neuronal progenitors have been active for several days before this. Do the authors consider that some defects are due to effects of losing UBE4B during early stages of cortical neurogenesis?

On page 11, please state in the text that panels 4E and H show P0 brains

Figure 6 shows a substantial increase in the number of cKO pups born after treatment with rapamycin, presumably as a result of restoring normal levels of neurogenesis during the last few days of embryonic development. It seems surprising that a minor decrease in the number of cortical (and/or dentate gyrus) neurons could cause prenatal death – could the authors comment on this?

A bit more detail is needed on exactly how cell counts were done in brain tissue – eg 4I, and 6, B-D, F,G and I – how many sections from how many animals? How was count area delineated?

In the discussion, the authors cite four papers that have previously also described the brain-specific deletion of UBE4 – did any of these studies find phenotypes consistent with those described here?

Reviewer 2

Advance summary and potential significance to field

In this manuscript the authors investigate the role of the E3/E4 ubiquitin ligase UBE4B in nervous system development. They generate mice carrying a new conditional knockout allele of UBE4B to delete expression in the nervous system using Nestin-Cre. Ube4b mutant mice have neurodevelopmental and behavioural phenotypes and die in the first few weeks of life. Using proteomics, they identity increased KLH22 levels, a regulator of the GATOR1 component DEPDC5, in Ube4b mutant mice brains and show that KLH22 is a substrate of UBE4B, suggesting that the phenotypes in Ube4b mutant mice may be due to mTOR activation. They show that mTOR activity is indeed increased in the developing brain in Ube4b mutant mice and that simultaneous knockout of KLH22 prevents mTOR activation.

Finally, they show that inhibition of mTOR signalling, using in utero treatment with rapamycin, abrogates mTOR activation and the neurodevelopmental defects in Ube4b mutant mice. This study demonstrates a novel neurodevelopmental role for UBE4B and links this to the extensive literature on the important role of mTOR signalling in neurodevelopment and neurological disease. This is an important finding for the field as it provides new mechanistic understanding of the mTOR pathway in neurodevelopment.

© 2022. Published by The Company of Biologists under the terms of the Creative Commons Attribution License (https://creativecommons.org/licenses/by/4.0/).

Comments for the author

The manuscript is very well written and has a clear logical structure. The figures are well presented and the statistical analyses are appropriate. The data mostly support the conclusions. I have the following comments:

1. The spatial expression pattern of UBE4B in the brain is analysed using RNAscope at P0, however Nestin-Cre is expressed from E10.5. It would be important to know the expression pattern of UBE4B during earlier stages of development, particularly in neurogenic regions such as the subventricular zone. Also, can the expression patten of UBE4B protein in the developing brain be analysed by immunohistochemistry?

2. The seizure phenotype of Ube4b mutant mice is shown in Fig S1E. These phenotypes would be much more convincing if videos of the mice were included.

3. The changes in FLAG-UBE4B levels shown in the left hand graph in Figure 3F are not described in the Results. The relevance of these data should be described.

4. In Supplemental Figure S4D pS6K levels should be normalised to total S6K levels.

5. The validation of the KLHL22 sgRNAs in vivo in Supplemental Figure 5C is not completely convincing. Can the authors validate the KLHL22 sgRNAs in cell culture?

6. Many mouse models of mTOR activation during neurodevelopment cause macrocephaly. Can the authors comment on why this was not the case in Ube4b mutant mice?

7. The authors state in the Abstract and elsewhere that mTOR inhibition rescues the neurodevelopmental defects in Ube4b mutant mice. The defects in NPC proliferation are shown in the dentate gyrus in Ube4b mutant mice in Figure 6 but the effects of rapamycin treatment are Sox2 expression are only shown in the forebrain in Figure 7G. It would support the claim that mTOR inhibition rescues the neurodevelopmental defects more strongly if it could be shown that rapamycin treatment rescues the neurogenesis defect in the dentate gyrus in Ube4b mutant mice.

8. The number of CTIP2+ GCs is reduced in Ube4b mutant mice until P30, when it is similar to controls. Can the authors suggest why this is? Is the level of apoptosis of CTIP2+ GCs reduced in Ube4b mutant mice at P30?

Minor comments

1. “Only a handful of UBE4B substrates have been identified, including the transcription factor p53.” This needs a citation.

2. Figure 1 legend: the final panel is described as (G) when it should be (I).

First revision

Author response to reviewers' comments

We thank the reviewers for their helpful comments and suggestions to improve and strengthen the manuscript. To address concerns raised by the reviewers, we have performed additional experiments and now include our new data and clarification in the revised manuscript. These changes are summarized here and described in detail in point-by-point response below.

- We include new data showing the spatial and temporal expression profiles of UBE4B during embryonic and neonatal stages (**Fig. 1A and 1B**).
- We include statistical analysis of pS6 levels normalized to total S6 levels (**Supplemental Fig. S4D6**).

© 2022. Published by The Company of Biologists under the terms of the Creative Commons Attribution License (https://creativecommons.org/licenses/by/4.0/).

- We include validation data of KLHL22 sgRNAs in Neuro2A cells (**Supplemental Fig. S5C**).
- We include quantification of PAX6$^+$ NPCs and DAPI-marked total cells in the DG from rapamycin-treated newborn *Ube4b* CKO animals (**Supplemental Fig. S7E-G**).
- We include a 4-minute video of an epileptic CKO mouse and its wild-type littermate as **Movie S1**.
- We include methodological clarifications as requested.
- We include modification and clarification in the Results and Discussion as requested.

Reviewer 1 Advance Summary and Potential Significance to Field.

Reviewer 1 found the paper provided “a significant advance” to the field and “the data presented are of high quality and are convincing. We thank the reviewer for his/her comments, and have addressed the concerns as follows:

Q: Fig 1 legend says that hippocampus in panel 1G is highlighted by red rectangles, these are missing on my copy.

We apologize for the mismatch between Figure 1G and its text. We have decided to remove red rectangles from Figure 1G as well as the relevant description in the Figure Legend.

Q: Analysis starts at E15.5, but many Nestin-expressing neuronal progenitors have been active for several days before this. Do the authors consider that some defects are due to effects of losing UBE4B during early stages of cortical neurogenesis?

We agree with the reviewer that some cortical defects might be possibly due to loss of UBE4B before E15.5, considering the cortical neurogenesis starts earlier than the hippocampal region. However, according to the only published knockout mouse model of UBE4B before our paper (Kaneko-Oshikawa et al. 2005), the protein expression of UBE4B was largely restricted to cardiac tissues and absent from the neural tube of wild-type embryos at E10.5. No obvious abnormalities in the overall morphology or cell apoptosis were observed in the brain region at E12.5 and E13.5 in this knockout model (Kaneko-Oshikawa et al. 2005). Thus, we suspect that UBE4B expression in the brain might be restricted at these stages.

In the revised manuscript, we have compared the expression of UBE4B in whole brains at E12.5 to those at E18.5 and P0. We have found that the protein and mRNA levels of UBE4B are indeed much lower at E12.5 than later stages. These data are now included in the revised manuscript as **Fig. 1A and 1B**. Meanwhile, our work has demonstrated that prenatal delivery of rapamycin starting at E15.5 rescues neurogenesis defects and perinatal lethality (**Fig. 7 and Supplemental Fig. S7**), suggesting that hyperactivation of mTOR by deletion of UBE4B may not cause irreparable damage to the brain before E15.5. Thus, we conclude that between E10.5-E15.5 UBE4B may not be as essential as its role at later stages.

Q: On page 11, please state in the text that panels 4E and H show P0 brains.

We thank the reviewer for the kind reminder. We have added the timing information to the text.

p. 11, “Immunohistochemistry of brain slices showed that deletion of UBE4B caused an induction of S6 phosphorylation in the dentate gyrus (DG) and cerebral cortex <u>of P0 brains</u> (Fig. 4E-H).”

Q: Figure 6 shows a substantial increase in the number of cKO pups born after treatment with rapamycin, presumably as a result of restoring normal levels of neurogenesis during the last few days of embryonic development. It seems surprising that a minor decrease in the number of cortical (and/or dentate gyrus) neurons could cause prenatal death - could the authors comment on this?

Between P0-P14 stages, immunohistochemistry of CITP2 showed that around 30-50% of neurons were lost in the DG region (**Fig. 6H-I**). Biochemistry analysis also revealed a great loss of neural progenitor cells and NeuN$^+$ neurons in P0 whole brains (Fig. 1H-I). At the behavioral level, UBE4B

© 2022. Published by The Company of Biologists under the terms of the Creative Commons Attribution License (https://creativecommons.org/licenses/by/4.0/).

CKO pups often died within a day after spontaneous seizures. Previous studies have showed that hyperactivation of mTOR caused by hippocampal deletion of other mTOR regulators can change synaptic E-I balance and cause lethal seizures (Bateup et al. 2013; Crino 2015). Thus, we consider the prenatal lethality is not surprising due to significant neuronal loss (our data) and possible E-I imbalance often observed in previous animal models of mTOR hyperactivation.

Q: A bit more detail is needed on exactly how cell counts were done in brain tissue - eg 4I, and 6, B-D, F, G and I. How many sections from how many animals? How was count area delineated?

We have added further method details in the Figure Legend and Materials and Methods as suggested. Counting areas have been outlined in indicated figures.

p. 44-45, "Phospho-S6 images from both groups were set to the same threshold. The ratio of pS6$^+$ CTIP2$^+$ cells in CTIP2$^+$ cells was calculated as the number of CTIP2$^+$ cells with pS6 signals above the threshold versus the total number of CTIP2$^+$ cells".

p. 25-26, "For quantification of NPC and GC numbers, the values were averaged across 9–15 brain sections per animal to cover the rostro and medial levels of the DG. The S1 cortex area above the DG in these brain slides was analyzed as well. For quantification of pS6 signal intensity, the average intensity of pS6 signals from cells across 6–9 brain slices in three pairs of control and CKO animals was analyzed. pS6 images from littermates were set to the same threshold. Cells with pS6 signals above the threshold were counted as pS6$^+$ cells. SOX2$^+$, Ki67$^+$, PAX6$^+$, CTIP2$^+$ and BrdU$^+$ cells were counted in the same way as pS6$^+$ cells. All quantitative analyses were performed with at least three pairs of wild-type and CKO animals."

Q: In the discussion, the authors cite four papers that have previously also described the brain-specific deletion of UBE4 - did any of these studies find phenotypes consistent with those described here?

We would like to clarify that, to our knowledge, the UBE4B mouse model in our work is the first brain-specific deletion model of UBE4B. The reviewer might have been misled by our discussion "Deletion of UBE4B in the nervous system caused phenotypes reassembling characteristics of mTORopathies (Liu and Sabatini 2020; Parenti et al. 2020), by which we meant to say that phenotypes of UBE4B CKO animals were similar to those observed in previous mutant mouse models of other mTOR regulators. We have revised this sentence to avoid confusion.

p. 16, "Deletion of UBE4B in the nervous system caused phenotypes reassembling characteristics of mTORopathies reported in mutant mouse models of other mTOR regulators (Liu and Sabatini 2020; Parenti et al. 2020).

Reviewer 2 Advance Summary and Potential Significance to Field.

Reviewer 2 found the manuscript was "very well written" and presented "an important finding for the field as it provides new mechanistic understanding of the mTOR pathway in neurodevelopment". We thank the reviewer for his/her comments, and have addressed his/her concerns as follows:

Q1: The spatial expression pattern of UBE4B in the brain is analyzed using RNAscope at P0, however Nestin-Cre is expressed from E10.5. It would be important to know the expression pattern of UBE4B during earlier stages of development, particularly in neurogenic regions such as the subventricular zone. Also, can the expression patten of UBE4B protein in the developing brain be analyzed by immunohistochemistry?

In the revised manuscript, we have further documented the spatial and temporal expression of UBE4B in E12.5, E18.5 and P0 brains parallelly by RNAscope. Indeed, as the reviewer predicted, UBE4B mRNAs are abundant surrounding lateral ventricles at embryonic and neonatal stages, which is consistent with its critical function in neurogenesis. These data are now included in the revised manuscript as **Fig. 1A**. In addition, we have found that the protein and mRNA levels of UBE4B are

© 2022. Published by The Company of Biologists under the terms of the Creative Commons Attribution License (https://creativecommons.org/licenses/by/4.0/).

much lower in E12.5 than later stages (**Fig. 1A and 1B**). This result is not surprising because previously Kaneko-Oshikawa et al reported that UBE4B expression was largely restricted to cardiac tissues and absent from the neural tube of wild-type embryos at E10.5. Furthermore, no obvious abnormalities in the overall morphology or cell apoptosis were observed in the brain region at E12.5 and E13.5 in their whole-body knockout mouse model of *Ube4b* (Kaneko-Oshikawa et al. 2005; Crino 2015). The restricted expression of UBE4B before E15.5 also explains our finding that prenatal delivery of rapamycin starting at E15.5 rescues neurogenesis defects in *Ube4b* CKO brains (**Fig. 7 and Supplemental Fig. S7**).

We agree with the reviewer that immunohistochemistry would be useful to demonstrate the spatial expression pattern of UBE4B in the brain. Indeed, we have previously tested available commercial antibodies (Thermo PA5-22023, HUABIO ET7111-11, and Abcam ab126759) and our home-made antibodies against UBE4B for immunocytochemistry and immunohistochemistry assays. Only the Abcam antibodies showed immunocytochemistry signals in cultured cells (validated by knockout cell lines). Unfortunately, none of them were suitable for immunohistochemistry experiments (no convincing difference between WT versus CKO brains). However, we hope that biochemistry and RNAscope data demonstrated in **Fig. 1, Supplemental Fig. S1 and Supplemental Fig. S4** would serve the purpose.

Q2: The seizure phenotype of Ube4b mutant mice is shown in Fig S1E. These phenotypes would be much more convincing if videos of the mice were included.

We have included a 4-minute video of an epileptic CKO mouse (on the left) and its wild-type littermate (on the right) as **Movie S1**. The original video was continuously recorded for 24 hours and the seizure episode in Movie S1 occurred at mid-night.

Q3: The changes in FLAG-UBE4B levels shown in the left-hand graph in Figure 3F are not described in the Results. The relevance of these data should be described.

We thank the reviewer for raising this important point. It is known that UBE4B is capable of self-ubiquitination and the U-box is essential for this process (Starita et al. 2013). Thus, deletion of the U-box will block its own degradation as well as the degradation of its substrates. We have discussed these data in the revised text.

p.10, “Further, we noticed that UBE4B Δbox also blocked its own degradation, which is consistent with previous knowledge that the U-box domain of UBE4B also mediates its auto-ubiquitination (Starita et al. 2013).”

Q4: In Supplemental Figure S4D pS6K levels should be normalised to total S6K levels.

We will add normalization data as **Supplemental Fig. S4D6** as suggested by the reviewer.

Q5: The validation of the KLHL22 sgRNAs in vivo in Supplemental Figure 5C is not completely convincing. Can the authors validate the KLHL22 sgRNAs in cell culture?

As the reviewer suggested, we have provided validation data of KLHL22 sgRNAs performed in Neuro2A cells as **Supplemental Fig. S5C**. Since the *in vivo* effect of sgRNAs also depends on the infection efficiency and protein turnover rate, it is reasonable that the apparent efficiency of sgRNAs was not as good as *in vitro* tests.

Q6: Many mouse models of mTOR activation during neurodevelopment cause macrocephaly. Can the authors comment on why this was not the case in Ube4b mutant mice?

We thank the reviewer for raising this interesting point. Indeed, we have commented on this phenomenon in the Discussion session. We hope the reviewer would agree on our explanation.

p. 16, “Deletion of UBE4B in the nervous system caused phenotypes reassembling characteristics of mTORopathies reported in mutant mouse models of other mTOR regulators (Crino 2015; Liu and Sabatini 2020; Parenti et al. 2020). However, the brain size and the thickness of cerebral cortex appeared normal in UBE4B deletion mice, with no signs of megalencephaly often observed in

© 2022. Published by The Company of Biologists under the terms of the Creative Commons Attribution License (https://creativecommons.org/licenses/by/4.0/).

animal models of mTOR hyperactivation. One possible explanation is that reduction in cell numbers had offset enlarged soma size, so that the brain volume remained largely unchanged in UBE4B CKO animals."

Q7: The authors state in the Abstract and elsewhere that mTOR inhibition rescues the neurodevelopmental defects in Ube4b mutant mice. The defects in NPC proliferation are shown in the dentate gyrus in Ube4b mutant mice in Figure 6, but the effects of rapamycin treatment are Sox2 expression are only shown in the forebrain in Figure 7G. It would support the claim that mTOR inhibition rescues the neurodevelopmental defects more strongly if it could be shown that rapamycin treatment rescues the neurogenesis defect in the dentate gyrus in Ube4b mutant mice.

We agree with the reviewer that analysis of neurogenesis in the DG region will further strengthen our conclusion. Thus, we have added quantification of $PAX6^{+}$ NPCs and DAPI-marked total cells in the DG as **Supplemental Fig. S7E-G** in the revised manuscript. Our new data have confirmed the reviewer's prediction that repamycin treatment also rescues the neurogenesis defect in the DG of Ube4B mutant mice.

Q8: The number of CTIP2+ GCs is reduced in Ube4b mutant mice until P30, when it is similar to controls. Can the authors suggest why this is? Is the level of apoptosis of CTIP2+ GCs reduced in Ube4b mutant mice at P30?

We thank the reviewer for raising this interesting point. Originally, we were also puzzled by the reduced difference between UBE4B CKO and control animals at P30. Indeed, two previous studies have documented that the number of neuronal cells quickly increases during the first one week in rats (Bandeira et al. 2009) or two weeks in mice (Lyck et al. 2007). Then the number of neurons will drop afterwards. It is known that this postnatal wave of neuronal apoptosis is an important manner to refine the neuronal network (Wong and Marin 2019). Our data also show that the number of neurons in the wild-type DG slightly dropped after P14, while no reduction of neurons was observed in the DG of CKO animals (**Fig. 7I**). Therefore, we agree with the reviewer's hypothesis that programmed neuronal death in UBE4B CKO animals may not be as vigorous as that in control animals, which may partially compensate the significant loss of NPCs and neurons caused by UBE4B deletion in the first two weeks.

Minor comment #1: "Only a handful of UBE4B substrates have been identified, including the transcription factor p53." This needs a citation.

We have added the citation as suggested by the reviewer.

p.8, "Only a handful of UBE4B substrates have been identified, including the transcription factor p53 (Antoniou et al. 2019)."

Minor comment #2: Figure 1 legend: the final panel is described as (G) when it should be (I).

We apologize for this error and have corrected the label in the Figure Legend.

Once again, we thank the reviewers for their helpful comments and suggestions, which have improved and strengthened the manuscript.

Zhiping Wang

References

Antoniou N, Lagopati N, Balourdas DI, Nikolaou M, Papalampros A, Vasileiou PVS, Myrianthopoulos V, Kotsinas A, Shiloh Y, Liontos M et al. 2019. The Role of E3, E4 Ubiquitin Ligase (UBE4B) in Human Pathologies. *Cancers (Basel)* **12**: 62-76.

Bandeira F, Lent R, Herculano-Houzel S. 2009. Changing numbers of neuronal and non-neuronal cells underlie postnatal brain growth in the rat. *Proc Natl Acad Sci U S A* **106**: 14108-14113.

© 2022. Published by The Company of Biologists under the terms of the Creative Commons Attribution License (https://creativecommons.org/licenses/by/4.0/).

Bateup HS, Johnson CA, Denefrio CL, Saulnier JL, Kornacker K, Sabatini BL. 2013. Excitatory/inhibitory synaptic imbalance leads to hippocampal hyperexcitability in mouse models of tuberous sclerosis. *Neuron* **78**: 510-522.

Crino PB. 2015. mTOR signaling in epilepsy: insights from malformations of cortical development. *Cold Spring Harb Perspect Med* **5**.

Kaneko-Oshikawa C, Nakagawa T, Yamada M, Yoshikawa H, Matsumoto M, Yada M, Hatakeyama S, Nakayama K, Nakayama KI. 2005. Mammalian E4 is required for cardiac development and maintenance of the nervous system. *Mol Cell Biol* **25**: 10953-10964.

Liu GY, Sabatini DM. 2020. mTOR at the nexus of nutrition, growth, ageing and disease. *Nat Rev Mol Cell Biol* **21**: 183-203.

Lyck L, Kroigard T, Finsen B. 2007. Unbiased cell quantification reveals a continued increase in the number of neocortical neurones during early post-natal development in mice. *Eur J Neurosci* **26**: 1749-1764.

Parenti I, Rabaneda LG, Schoen H, Novarino G. 2020. Neurodevelopmental Disorders: From Genetics to Functional Pathways. *Trends Neurosci* **43**: 608-621.

Starita LM, Pruneda JN, Lo RS, Fowler DM, Kim HJ, Hiatt JB, Shendure J, Brzovic PS, Fields S, Klevit RE. 2013. Activity-enhancing mutations in an E3 ubiquitin ligase identified by high-throughput mutagenesis. *Proc Natl Acad Sci U S A* **110**: E1263-1272.

Wong FK, Marin O. 2019. Developmental Cell Death in the Cerebral Cortex. *Annu Rev Cell Dev Biol* **35**: 523-542.

Second decision letter

MS ID#: DEVELOP/2022/201286

MS TITLE: Fine-tuning of mTOR signaling by the UBE4B-KLHL22 E3 ubiquitin ligase cascade in brain development

AUTHORS: Xiangxing Kong, Xin Shu, Jiachuan Wang, Dandan Liu, Yingchun Ni, Weiqi Zhao, Lebo Wang, Zhihua Gao, Jiadong Chen, Bing Yang, Xing Guo, and Zhiping Wang
ARTICLE TYPE: Research Article

I am delighted to tell you that your manuscript has been accepted for publication in Development, pending our standard ethics checks.

Reviewer 2

Advance summary and potential significance to field

I am satisfied with the modifications to the revised manuscript and now recommend publication.

Comments for the author

I am satisfied with the modifications to the revised manuscript and now recommend publication.

© 2022. Published by The Company of Biologists under the terms of the Creative Commons Attribution License (https://creativecommons.org/licenses/by/4.0/).
